# The UlaG protein family defines novel structural and functional motifs grafted on an ancient RNase fold

**DOI:** 10.1186/1471-2148-11-273

**Published:** 2011-09-26

**Authors:** Francisco J Fernandez, Fernando Garces, Miguel López-Estepa, Juan Aguilar, Laura Baldomà, Miquel Coll, Josefa Badia, M Cristina Vega

**Affiliations:** 1 Structural and Quantitative Biology Department, Centro de Investigaciones Biológicas (CIB-CSIC), Ramiro de Maeztu 9, 28040 Madrid, Spain; 2 Genome Damage and Stability Centre, University of Sussex, Falmer, Brighton, BN1 9RQ, UK; 3 Departament de Bioquímica i Biologia Molecular, Institut de Biomedicina de la Universitat de Barcelona (IBUB), Facultat de Farmàcia, Universitat de Barcelona, 08028 Barcelona, Spain; 4 Institut de Biologia Molecular de Barcelona (IBMB-CSIC), Barcelona Science Park, Baldiri Reixac 10, 08028 Barcelona, Spain; 5 Institute for Research in Biomedicine (IRB Barcelona), Barcelona Science Park, Baldiri Reixac 10, 08028 Barcelona, Spain

## Abstract

**Background:**

Bacterial populations are highly successful at colonizing new habitats and adapting to changing environmental conditions, partly due to their capacity to evolve novel virulence and metabolic pathways in response to stress conditions and to shuffle them by horizontal gene transfer (HGT). A common theme in the evolution of new functions consists of gene duplication followed by functional divergence. UlaG, a unique manganese-dependent metallo-β-lactamase (MBL) enzyme involved in L-ascorbate metabolism by commensal and symbiotic enterobacteria, provides a model for the study of the emergence of new catalytic activities from the modification of an ancient fold. Furthermore, UlaG is the founding member of the so-called UlaG-like (UlaGL) protein family, a recently established and poorly characterized family comprising divalent (and perhaps trivalent) metal-binding MBLs that catalyze transformations on phosphorylated sugars and nucleotides.

**Results:**

Here we combined protein structure-guided and sequence-only molecular phylogenetic analyses to dissect the molecular evolution of UlaG and to study its phylogenomic distribution, its relatedness with present-day UlaGL protein sequences and functional conservation. Phylogenetic analyses indicate that UlaGL sequences are present in Bacteria and Archaea, with bona fide orthologs found mainly in mammalian and plant-associated Gram-negative and Gram-positive bacteria. The incongruence between the UlaGL tree and known species trees indicates exchange by HGT and suggests that the UlaGL-encoding genes provided a growth advantage under changing conditions. Our search for more distantly related protein sequences aided by structural homology has uncovered that UlaGL sequences have a common evolutionary origin with present-day RNA processing and metabolizing MBL enzymes widespread in Bacteria, Archaea, and Eukarya. This observation suggests an ancient origin for the UlaGL family within the broader trunk of the MBL superfamily by duplication, neofunctionalization and fixation.

**Conclusions:**

Our results suggest that the forerunner of UlaG was present as an RNA metabolizing enzyme in the last common ancestor, and that the modern descendants of that ancestral gene have a wide phylogenetic distribution and functional roles. We propose that the UlaGL family evolved new metabolic roles among bacterial and possibly archeal phyla in the setting of a close association with metazoans, such as in the mammalian gastrointestinal tract or in animal and plant pathogens, as well as in environmental settings. Accordingly, the major evolutionary forces shaping the UlaGL family include vertical inheritance and lineage-specific duplication and acquisition of novel metabolic functions, followed by HGT and numerous lineage-specific gene loss events.

## Background

Bacteria can evolve complex virulence mechanisms and metabolic pathways by gene duplication and functional divergence of one of the copies [1,2], with the ancestral copy retaining the original function, and by shuffling these entire pathways by horizontal gene transfer (HGT) [3,4]. Indeed, gene duplication has been presented as a major driving force behind evolutionary change [1,2], with 70% and 90% of duplicated domains found in bacteria and eukaryotic organisms, respectively [5]. However, the relative impact of gene duplication and HGT in the generation of genetic diversity and their role in adaptation is still under debate [3]. The opposite process of gene loss has been hypothesized to favor the evolution of prokaryotes under intermittent selective pressures (coupled to the regain of function in the form of cryptic genes, such as those involved in β-glucoside degradation found in most natural isolates of *E. coli*) [6,7]. Other examples of gene loss occur in response to lifestyle adaptations like the dramatic genome reduction of obligate host-associated bacteria such as *Mycoplasma *and *Buchnera *[8], or to allow for more efficient replication in free-living microorganisms like in the marine free-living bacteria *Prochlorococcus *and *Pelagibacter *[9,10]. The prevalence of HGT in bacteria, especially in multi-species ecological communities such as those in the human gastrointestinal tract, plays an essential role in generating genetic variation in addition to gene duplication and gene loss [11]. HGT events are crucial for the dissemination of beneficial functional traits between bacterial communities especially when these are under strong selection pressure, as is the case for virulence factors among pathogenic bacteria [12,13].

A number of protein folds have been extensively re-used during evolution for the emergence of novel functions, including metallo-β-lactamase (MBL) [14], triose-phosphate isomerase (TIM) [15,16], and Rossmann fold scaffolds [15], in particular in the context of metabolism, which plays a crucial role in bacterial adaptation as shown by the massive expansion of metabolic superfamilies since the last universal common ancestor (LUCA) [17]. One of the underlying reasons for the success of these folds in acquiring new functions is that they hold a wide active site that can accommodate complex and multiple substrates simultaneously and the presence of many surrounding loops, modifications of which can alter the chemical and catalytic properties of the protein, its substrate/product profile, or cofactor-binding properties. MBLs provide an important source of versatile catalysts that have an ancient evolutionary origin and a widespread phylogenomic distribution across Bacteria, Archaea, and Eukarya [14]. The central role of MBLs in both cellular and metabolic housekeeping functions as well as in the rapid spread of multi-drug resistant pathogens calls for a better understanding of the evolutionary mechanisms by which novel functions evolve from an archetypical protein fold.

UlaG, a recently characterized MBL sequence with a novel function and unique sequence and structural features [18,19], is a protein encoded by a gene from the L-ascorbate utilization (*ula*) regulon of Enterobacteria [20]. The *ula *regulon consists of two divergently transcribed units (Additional file 1). The first is the *ula*G cistron that encodes the L-ascorbate 6-phosphate lactonase (UlaG) involved in the conversion of L-ascorbate 6-phosphate to L-gulonate 6-phosphate in the catabolism of L-ascorbate [18] (Additional file 2). The second unit comprises the *ula*ABCDEF operon. Genes *ula*ABC encode the transmembrane phosphotransferase system (PTS) responsible for the uptake of L-ascorbate from the medium in the form of L-ascorbate 6-phosphate. The structural genes *ula*D (3-keto-L-gulonate 6-phosphate decarboxylase), *ula*E (L-xylulose 5-phosphate 3-epimerase), and *ula*F (L-ribulose 5-phosphate 4-epimerase), act sequentially on the product of UlaG to convert it to D-xylulose 5-phosphate, an intermediate of the pentose phosphate pathway (Additional file 2). The *ula *regulon is under transcriptional control of *ula*R, located upstream to *ula*G. Expression of the structural genes of the *ula *regulon is controlled by changes in the quaternary architecture of UlaR, a DeoR-family repressor, which, in the absence of extracellular L-ascorbate, represses transcription of the regulon by binding DNA as a homotetrameric complex to four operator sequences (Additional file 1). The availability of L-ascorbate triggers transcriptional activation by binding of the inducer molecule L-ascorbate 6-phosphate to UlaR, thereby leading to its concomitant conversion to a DNA-free dimeric complex that can no longer maintain a repressed state [21].

**Figure 1:**
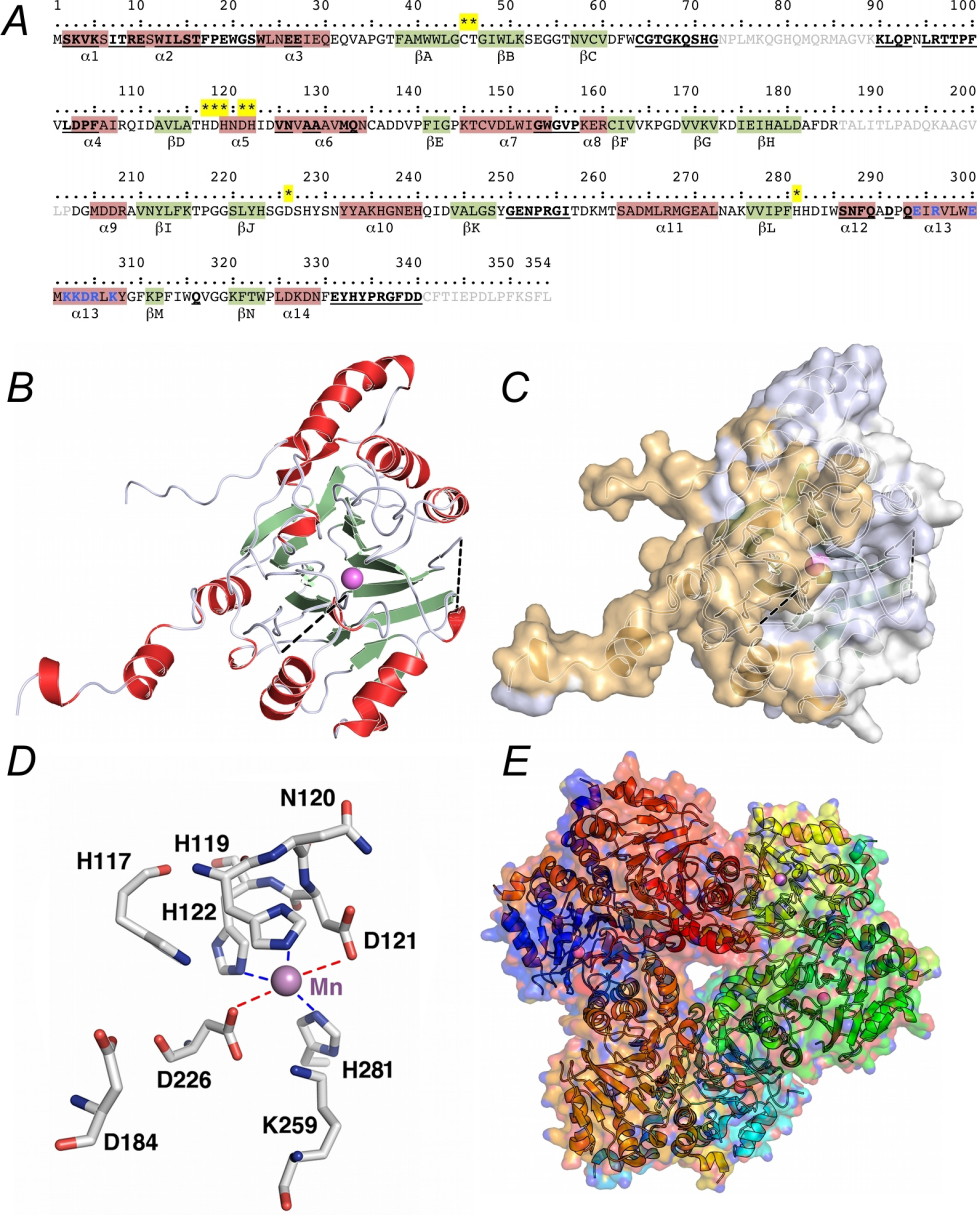
**Structure and sequence motifs of the founding member of the UlaG protein family, *E. coli *L-ascorbate 6-phosphate lactonase**. *A*. Primary sequence of UlaG decorated with structural (from the Mn^2+^-loaded crystal structure, PDB 2wym) and functional annotations. Sequence segments not visible in the experimental electron density are shaded in gray. Secondary structure elements are shown as background colors, red (α-helices) and green (β-strands), and labeled from α1-α14 and βA-βN. Residues that form part of interaction surfaces are in bold and underlined. Finally, residues that directly (or indirectly) contribute to the Mn^2+^-binding site are marked with a star on a yellow background. *B*. Monomer structure of UlaG determined by X-ray crystallography at 2.6-Å resolution (PDB 2wym). Dashed lines represent missing loops in electron density (residues shaded in gray in *A*). *C*. Surface representation of the monomer where the interfacing residues are shown in light orange and are mapped onto the surface. *D*. Mn^2+^-binding site of UlaG. Side chains of residues from the coordination sphere are shown as sticks in atom colors and liganded Mn^2+ ^ion in violet. *E*. Hexameric arrangement characteristic of the biologically active UlaG enzyme viewed along the threefold molecular symmetry axis.

Of the L-ascorbate catabolic genes, only the catalytic activity of the *ula*G gene product has not been confidently assigned on the basis of sequence homology, and it was not until last year [18] that the activity of UlaG was established experimentally. In addition, the determination of the crystal structure of UlaG at 2.6-Å resolution [18] corroborated sequence-based predictions that the fold was similar to Zn^2+^-dependent hydrolases of the MBL superfamily while simultaneously revealing several unforeseen properties (Figure 1A-E). First, the UlaG fold resembles that of MBL RNases with additional conspicuous adaptations to its new functional role in L-ascorbate catabolism (Figure 1B and 1C). Second, it is the first characterized Mn^2+^-dependent MBL (Figure 1D). And, third, UlaG has a distinctive oligomeric structure (a hexamer) that had not been previously described for MBLs (Figure 1E). Prompted by these unusual properties of UlaG, which suggested that the ancestral RNase fold of this protein had been subjected to extensive modifications in order to acquire new catalytic activity and regulatory mechanisms in a completely different ecological scenario [15], we set out an in-depth study to characterize the protein family founded by UlaG. In this study we present the molecular evolution of the UlaG protein family, its phylogenomic distribution, and genome context, and we construct an evolutionary pathway from the ancestral RNase fold to present-day UlaG-like proteins in prokaryotic organisms.

## Results

### Phylogenetic analysis

**Figure 2:**
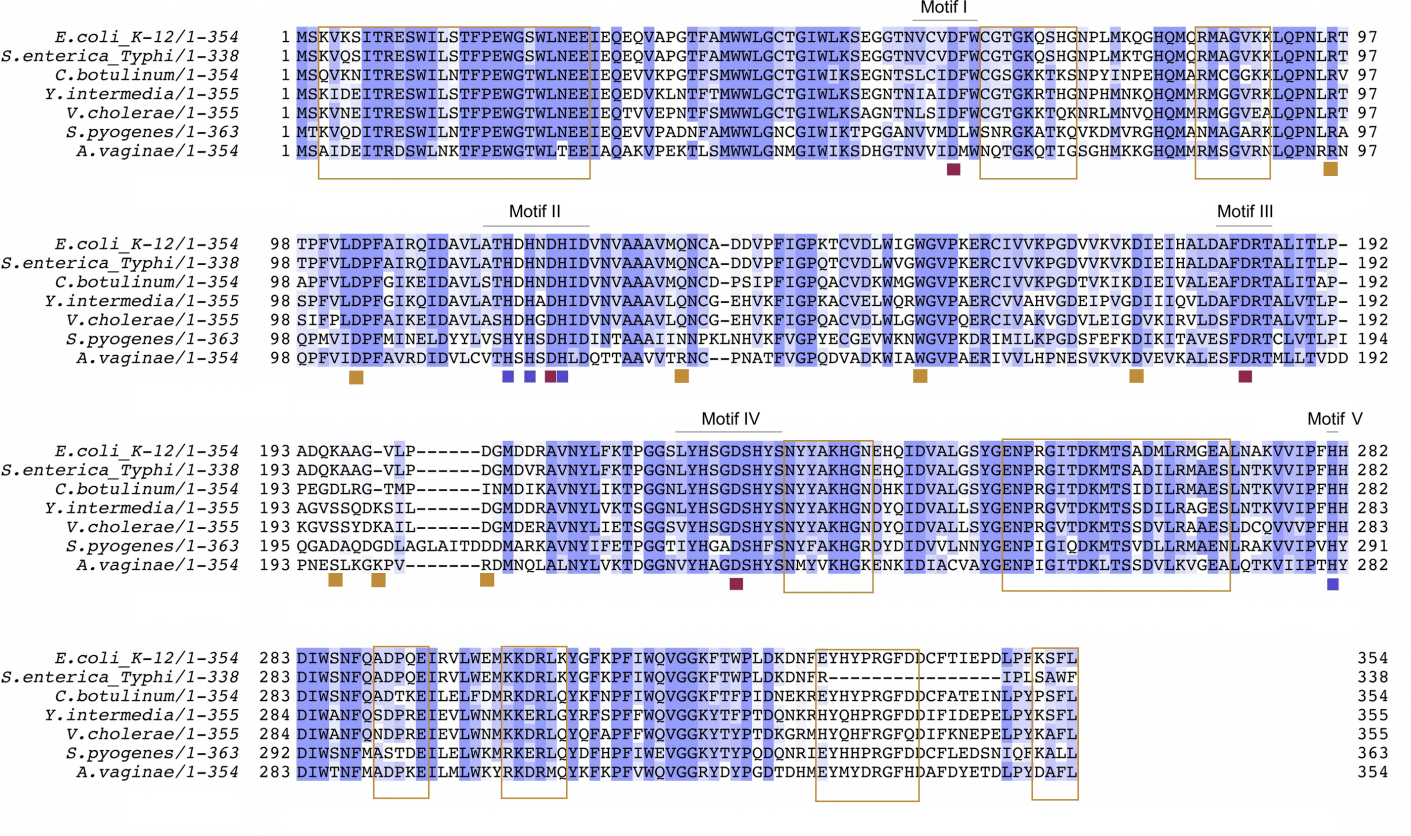
**Multiple sequence alignment of UlaGL protein sequences among bacteria**. Multiple sequence alignment of *E. coli *UlaG (*E. coli*; top sequence) against six divergent UlaGL sequences from Gram-negative and Gram-positive bacteria. In descending order, sequences included are putative L-ascorbate 6-phosphate lactonases from *Salmonella enterica *subsp. *enterica *serovar Typhi str. CT18 (NP_458816); *Klebsiella pneumoniae *subsp. *pneumoniae *MGH 78578 (YP_001338203); *Clostridium botulinum *B str. Eklund 17B (YP_001887453); *Yersinia intermedia *ATCC 29909 (ZP_046368); *Vibrio cholerae *V51 (ZP_04919573); *Streptococcus pyogenes *MGAS315 (NP_663946); and *Atopobium vaginae *DSM 15829 (ZP_039469). Pairwise sequence identities are larger than 50% for all sequences in the multiple sequence alignment. Characteristic motifs of the metallo-β-lactamase protein fold are shown underlined on top of the alignment, and absolutely conserved catalytic aspartate and histidine residues within these motifs are marked underneath the alignment as red or blue squares, respectively. Residues involved in intersubunit contacts are boxed.

Sequence similarity searches with *E. coli *UlaG primary sequence [Genbank:P39300] against a database of non-redundant protein sequences revealed that proteins with sequence identity greater than 50%, and therefore possibly homologous to UlaG, are present in bacterial genomes belonging to three eubacterial phyla across several classes and families (Figure 2; Additional files 3 and 4). Bacterial lineages containing at least one potential UlaG homolog, or UlaG-like (UlaGL) sequence, include families from the divisions *Proteobacteria *(*Enterobacteriaceae *genera *Escherichia*, *Salmonella*, *Shigella*, *Citrobacter*, *Enterobacter*, *Klebsiella*, *Yersinia*, *Providencia*, *Actinobacillus*, *Haemophilus*, *Mannheimia*, and *Pasteurella*; *Vibrionales *genera *Vibrio *and *Photobacterium*); *Firmicutes *(*Clostridiales *genus *Clostridium*; *Ruminococcaceae *genera *Anaerotruncus*, *Ruminococcus*, and *Epulopiscium*; *Lactobacillales *genera *Enterococcus*, *Lactobacillus*, *Leuconostoc*, and *Streptococcus*) and *Actinobacteria *(*Coriobacteriaceae Atopobium*). These genera include commensal and symbiotic bacteria, opportunistic pathogens, and pathogenic bacteria that colonize the mammalian gastrointestinal tract (GIT), oral mucosa, genitourinary tract, as well as bacteria found in environmental reservoirs such as soil and water.

**Figure 3:**
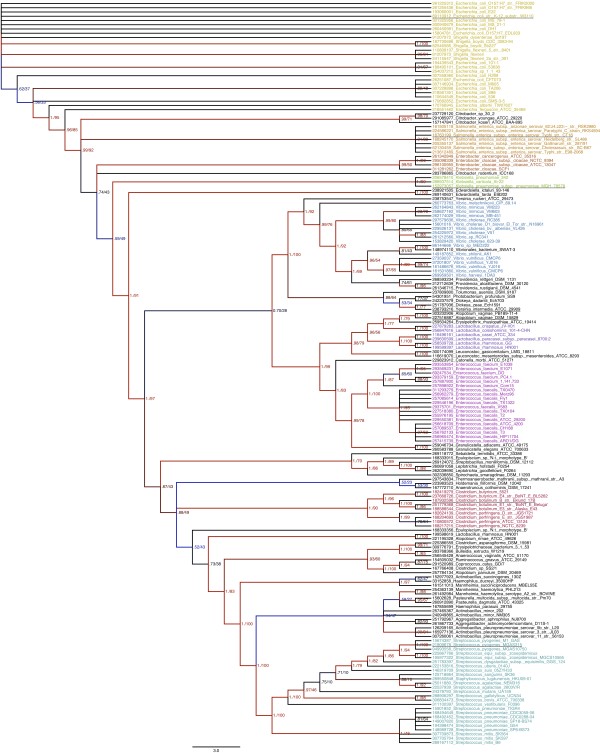
**Bayesian inference tree of UlaGL orthologs from 330 aligned amino acid positions**. The consensus tree was produced with FigTree from the last 9,500 trees representing 950,000 stationary generations. Bayesian inference posterior probability (BIPP) and maximum-likelihood bootstrap percentage (MLBP) are indicated on nodes. Only branches with > 0.50 BIPP support are labeled with BIPP and MLBP. The SDSF at the end of the MrBayes run was 0.008. The scale bar below the tree shows the evolutionary distance expressed as substitutions per site. Taxon names are prefixed with their NCBI GI numbers. The underlined taxa are shown in the representative alignment (Figure 2). Nexus alignment files are available from the authors on request.

Comparison of unrooted phylogenetic trees constructed from curated multiple sequence alignments of UlaGL sequences (Figure 3; Additional files 5, 6 and 7) with the known phylogeny of *Proteobacteria *and Gram-positive bacteria [22-24] allows us to tentatively place the last common ancestor around the radiation of the *Firmicutes *from the common branch that would later split into *Proteobacteria *and *Actinobacteria*. Interestingly, the first radiation event in the phylogenetic tree of the UlaG homologs coincides with the emergence of class B3 β-lactamases, prior to the split of *Proteobacteria *from Gram-positive bacteria [25]. This observation is consistent with the proposed evolutionary origin of MBL sequences without β-lactamase activity from an ancient, essential protein, such as an RNA-processing enzyme [14,25].

The analysis of the UlaGL gammaproteobacterial subtree (a tree containing solely gammaproteobacterial sequences) and its comparison to the corresponding species tree (Additional file 8) using the Shimodaira-Hasegawa test supports a model of near exclusive vertical transmission (*p *= 1.0 at 5% confidence level). In contrast, neither the *Firmicutes *nor the *Actinobacteria *branches are completely orthologous groups. The *Actinobacteria*, represented by *Atopobium *spp., appears interspersed between proteobacterial and *Firmicutes *lineages, while the *Firmicutes *branches contain sequences from enterobacterial species (e.g., *Yersinia *spp., *Dickeya *spp., and *Mannheimia *spp.). The incongruent placement of enterobacterial sequences within Gram-positive clades indicates that some HGT events between enterobacteria and bacteria from other taxonomic groups within these categories have occurred. This possibility has already been proposed for the MBL superfamily [25]. These observations suggest a far more recent origin for *ula*GL in the common ancestor to *Vibrionales*, *Enterobacteriales *and *Pasteurellales*. In this scenario, the absence of an UlaGL protein in distantly related *Gammaproteobacteria *(e.g., *Pseudomonas*, *Xanthomonas *or *Xylella*) would be a natural consequence of the *ula*GL birth after the split of their common ancestor. In addition, the current distribution of *ula*GL within *Firmicutes*/*Actinobacteria *would require only a very reduced number of HGT events with an enterobacterial species as donor and a Gram-positive bacterium as recipient. A case in point is the presence of *Pasteurella *and *Mannheimia *UlaGL nested inside the *Clostridium *and *Streptococcus *clades; this tree topology can be explained by one HGT event from a *Pasteurelalles *ancestor to the common ancestor of (*Clostridiales *+ *Streptococcales*) or several later HGT events to each individual recipient lineage.

For most prokaryotic species, we have identified a single UlaG homolog per genome (Additional file 3). Indeed, we detected only 4 species with more than one UlaG homolog (above an E-value threshold of 1 × 10^-10^). This finding thus indicates the existence of paralogous sequences in restricted cases. Three such species are eubacterial, including *Epulopiscium *sp. 'N.t. morphotype B' (2 paralogs), *Dickeya zeae *Ech159 (2 paralogs), and *Sebaldella termitidis *(2 paralogs), and one is archeal, *Haloterrigena turkmenica *DSM 5511 (3 paralogs). In *Epulopiscium *sp. 'N.t. morphotype B' the two sequences (gi|168333356 and gi|168333915) are annotated as hypothetical proteins, and the first sequence lacks similarity to the first 18 amino acids of UlaG, thus the N-terminal hexamerization motif has been weakened or abolished. The two paralogous sequences of *Dickeya zeae *Ech1591 (ref|YP_003003802.1 and gb|ACT06323.1) are also listed as hypothetical proteins. In contrast, the UlaGL paralogs in *S. termitidis *are annotated as L-ascorbate 6-phosphate lactonases and are part of a near complete operon duplication. Except for sequencing or annotation error, the two UlaGL sequences in *S. termitidis *share 100% sequence identity, which is consistent with a very recent duplication event.

Although RNA processing enzymes did not appear to have generally been captured by a sequence-only search, at least one such sequence was present in iteration 1 of PSI-BLAST. It corresponds to a putative mRNA 3'-end processing factor from the Crenarcheota *Pyrobaculum islandicum *DSM 4184. The length of the alignment is limited (about 100 residues) but in this limited length it contains 33% sequence identity and 48% similarity, and contains similarity to the amino-acid stretch spanning 96-182, a region that includes the central portion of the active site and adjacent elements. This degree of sequence conservation places this hit slightly above the twilight zone of hard-to-establish similarity, and, in conjunction with the structural homology to MBL RNases, provides further evidence of a distant evolutionary relation of the UlaG family with MBL RNases.

Finally, we detected a series of sequences corresponding to the *romA *gene encoding for a multi-drug resistance membrane protein and bearing some degree of sequence homology to UlaG. However, the low sequence identity (always below the 30% identity threshold; best hits had 18% identity, 35% similarity, and 350-360 aligned residues, with a bit score of 327 and a E-value of 10^-87^) makes this link less reliable. However, it is not inconceivable that the UlaG and the RomA proteins had an ancient common ancestor and that their markedly different functions arose by divergence over a very long evolutionary time.

### Conservation of UlaG features among orthologous sequences

UlaG has several distinctive motifs that confer its characteristic structure and support its catalytic activity and that are expected to be conserved among putative orthologous sequences. Aside from the metal-binding motif and the presence of long loops over the active site, presumably for substrate binding and assisting catalysis, which are also found in more distantly related MBLs, the most conspicuous features of UlaG are found in its amino (N) and carboxy (C) termini. The N terminus spans thirty residues and folds as three consecutive α-helices (Figure 1A and 1B). Helices α1 and α2 form a mostly positively charged extended arm that inserts into a complementary, negatively charged valley made from helix α3, thereby suggesting that the conservation of this oligomerization motif requires the conservation of the complementary features of helices α1-α2 and α3. Truncations of the two first α-helices α1 and α2 are expected to weaken the greatest contact area between adjacent UlaG subunits and, accordingly, would destabilize a putative hexameric complex. Likewise, though less dramatically, C-terminal truncations deleting residues 331-340, located immediately after the last α-helix, α14, would be expected to remove contact area residues and therefore compromise the oligomeric structure. Indeed, of all the 180 bona fide UlaG orthologs only in *Shigella dysenteriae *Sd197 the N-terminal helices α1-α2 are lacking. The only other orthologous sequence with an incomplete N terminus, *Mannheimia haemolytica *PHL213 UlaG, appears to lack helix α1. However, it is uncertain whether the apparent loss is reliable or caused by a sequencing artifact. Interestingly, the presence of additional N-terminal residues is observed more often than the loss of the N-terminal helical motif. In 53 out of 180 sequences (29.4%), the N terminus contains additional residues when compared to the *E. coli *UlaG reference sequence. Of these, 15 sequences (28%) have N-terminal extensions longer than 5 residues (the longest having 31 amino acids) and most (14 or 92%) have not lost a methionine residue at the reference position of Met-1. Since all these sequences are uncharacterized, it is still unclear whether the predicted initiation codon is used or whether the reference reading frame is used instead. In support of the correct assignment of the predicted initiation codons, virtually all these sequences contain 1-3 lysine residues, which might play a similar role to the two lysine residues in helix α1.

The C-terminal end of *E. coli *UlaG contains elements essential for the oligomerization and for organization of the channel-like architecture of the hexameric molecule (Figure 1, A, 1C and 1E). Residues 285-354 encompass helices α12 and α13, loop βM-βN, and a 10-amino-acid stretch following helix α14, all of which include interface residues. Helix α13 occupies a central location in the UlaG molecule and is hypothesized to provide further stabilization to the quaternary structure by the interactions it makes with the symmetrical helices in the other subunits. The crucial role of the C terminus is underlined by the fact that only three sequences among the orthologous set show considerable deviations from the consensus sequence. Two of these sequences (cattle pathogenic *Escherichia coli *O157:H7 str. FRIK2000 and FRIK966) lack a stretch from helix α14 to the C terminus, apparently as a result of the mutation of a tryptophan TGG codon (Trp-323 in *E. coli *UlaG) to a stop TAG codon. The third sequence, from *Salmonella enterica *subsp. *enterica *serovar Typhi str. E98-2068, has specifically lost the ten amino-acid residues involved in intersubunit contacts while it has retained helix α14 as well as the last 7 amino acids.

### Highest variable regions among closely related sequences

Among highly similar regions, for example, across different *E. coli *strains, single amino-acid substitutions tend to be conservative substitutions of small hydrophobic or polar uncharged residues in or preceding β-strands, including positions in the neighborhood of the active site. This observation is not surprising since small hydrophobic residues in β-strands frequently have structural support roles that do not cause changes in function. As for α-helices, for example, Met-301 in α13 is conservatively replaced by an isoleucine residue in *Shigella flexneri *UlaG, where it is the only amino-acid change along the entire length of the sequence. In this particular case, the side chain of Met-301 points to the solvent channel in the middle of the UlaG hexamer and is unlikely to carry any critical functional role in stability or catalysis.

Inspection of hyper-variable regions between closely related enterobacterial sequences shows that most of the variation does cluster in two α-helices, α5 (spanning residues 145-154) and α7 (262-271). In both cases, the mutations are conservative and should not disrupt the structural elements surrounding them. The α5 helix is fully exposed and its C terminus establishes helix-helix dipolar interactions with the short α1 helix. In contrast, α7 is part of the second largest interface in the quaternary structure of UlaG, where it forms part of two symmetrical α-helical bundles with an extensive surface interaction.

As soon as sequence identity falls below 80%, substitutions appear in and around the active site and metal-binding loops, suggesting that there might be shifts in substrate specificity or reactivity. In UlaG homologous sequences found in various *Clostridium *sp. including human pathogenic strains with less than 80% sequence identity (74-76%), all metal-binding ligands are conserved against a background of mostly conservative, evenly distributed mutations. This indicates that the overall structure has been preserved despite the possibility that the active site loops accumulated enough changes to have shifted in substrate specificity.

### Phylogenetic analysis of prokaryotic halophilic UlaGL

**Figure 4:**
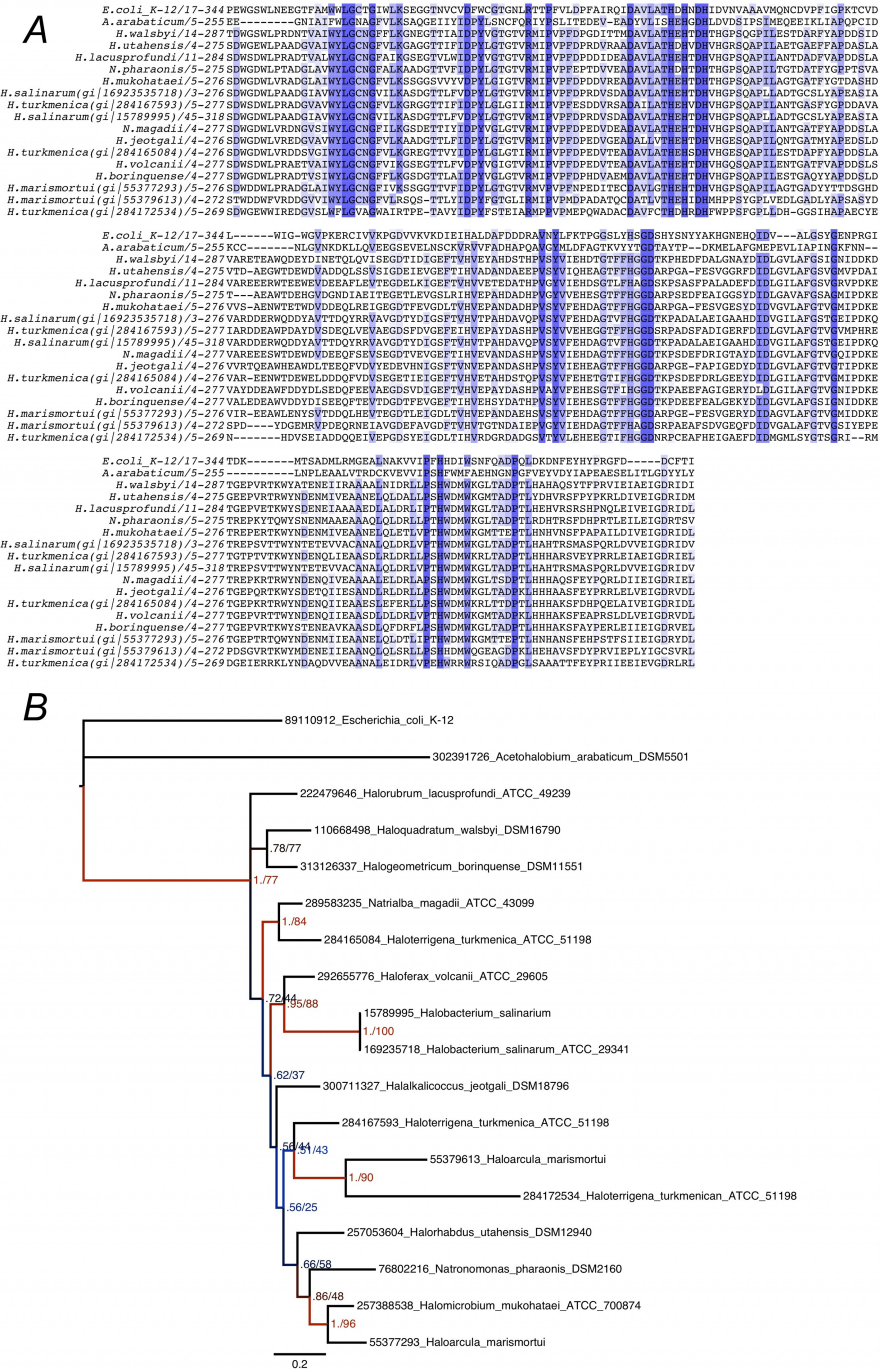
**Horizontal gene transfer of UlaGL genes to halophilic Archea**. *A*. Alignment of UlaGL sequences from halophilic archea, along with two reference sequences (*E. coli *UlaG, and the halophilic bacterium *Acetohalobium arabaticum*). *B*. Bayesian consensus tree of the same sequences in *A*, with *E. coli *UlaG as the outgroup. Bayesian inference posterior probability (BIPP) and maximum-likelihood bootstrap percentage (MLBP) are indicated on nodes. Only branches with > 0.50 BIPP support are labeled with BIPP and MLBP. The SDSF at the end of the MrBayes run was 0.007. The scale bar below the tree shows the evolutionary distance expressed as substitutions per site. Taxon names are prefixed with their NCBI GI numbers. Nexus alignment file is available from the authors on request.

The closest non-eubacterial UlaGL sequences are present in Archaea from Euryarcheota and display 26% sequence identity (42% similarity) with the top aligned UlaGL sequences (Figure 4A; Additional files 9, 10 and 11). These archeal sequences appear to be restricted to several extremophilic species of the *Halobacteriaceae *family (Figure 4), including organisms such as *Haloterrigena turkmenica *DSM 5511, *Haloquadratum walsbyi *DSM 16790, *Halorhabdus utahensis *DSM 12940, *Natronomonas pharaonis *DSM 2160, *Halogeometricum borinquense *DSM 11551, *Halobacterium *sp. NRC-1, *Halobacterium salinarum *R1, *Haloferax volcanii *DS2, *Halomicrobium mukohataei *DSM12286, *Natrialba magadii *ATCC 43099, *Halorubrum lacusprofundi *ATCC 49239, *Halalkalicoccus jeotgali *B3, and *Haloarcula marismortui *ATCC 43049. This severely restricted ecological distribution of the archeal UlaGL sequences suggests that the most parsimonious evolutionary scenario for their origin is an HGT event from a eubacterial species. In support of this hypothesis, the eubacterial halobacterium *Acetohalobium arabaticum *DSM 5501, which thrives under similar environmental conditions as the *Halobacteriaceae*, shares with this family the same protein sequence, which shows a similar degree of conservation with the cluster of UlaGL sequences (24-26%). Furthermore, phylogenetic trees reconstructed from alignments of the archeal sequences and the *E. coli *and *A. arabaticum *UlaGL sequences provide support for a tight clustering in the origin and diversification of the halophilic archeal UlaGL sequences, with multiple duplication events in various lineages (including *Haloterrigena turkmenica*, *Halobacterium salinarum*, and *Haloharcula marismortui*) (Figure 4B; Additional file 12). In agreement with the proposal that these archeal UlaGL sequences are functionally unrelated to the remaining eubacterial UlaGLs, they all conserve a core sequence spanning approximately 275 residues that lacks two critical regions for UlaG oligomerization, the N-terminal α1-α2 motif and α13 (the central channel helix) and βM-βN, as well as 20-amino-acid segment of the loop βC-β4 following catalytic motif I (Figure 1 and 4A). The absence of the extreme N- and C-terminal sequences that are involved in shaping the characteristic hexameric structure of UlaG, suggests that the haloextremophile sequences, both archeal and eubacterial, do not assemble into hexameric structures.

### Conservation of the *ula *regulon structure and genomic context

**Figure 5:**
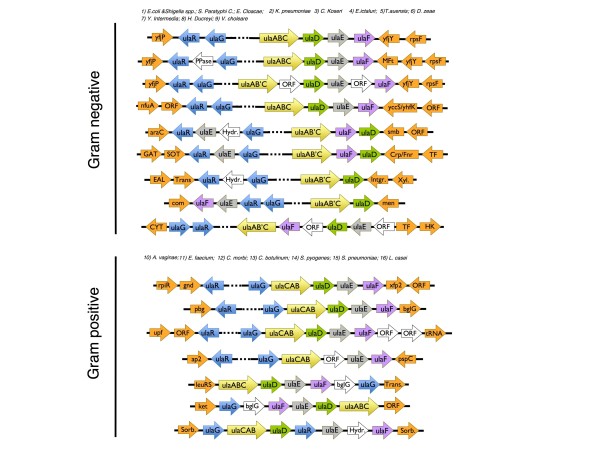
**Genome context of UlaG in bacterial genomes**. True orthology between similar sequences often requires conservation of the gene's genomic context, especially in the context of gene clusters encoding for signal transduction cascades or metabolic pathways. The genome context around *ula*G-like genes in >100 bacterial genomes (including many genomes from human symbiotic bacteria and pathogens) was examined. For clarity, only a representative set of consistent genomic arrangements is shown. Arrows represent the gene order and orientation of the transcription of each per species. Dashed lines connecting arrows represent larger intergenic spaces accommodating transcription regulatory sequences. Relevant taxonomic groupings are shown.

It has been proposed that the organization of genes into operons is the main reason for conservation of adjacency in prokaryotic genomes [26]. The genomic context can provide insight into function, since functionally related genes tend to be contiguous and co-transcribed in bacteria [22]. Accordingly, we searched the genomes that encode proteins with significant sequence identity to UlaG (putative orthologs, with sequence identity greater than 55%) to study whether the homologous gene was part of a similar regulon structure to that found in *E. coli*. As a straightforward method, we first searched for UlaR homologous proteins whose respective genes would be adjacent to those encoding the corresponding UlaG homologs. Indeed, a *ula*R homologous gene was found adjacent to each *ula*G homologous gene in all Gram-negative and Gram-positive genomes examined except for the phylogenetically-isolated Fusobacterium *Sebaldella termitidis *ATCC 33386 [27] and *Streptococcus *spp., where a gene encoding a BglG transcription regulator was found next to the respective *ula*G instead of *ula*R encoding a DeoR-family repressor (Figure 5). Proteins belonging to the BglG family are bacterial RNA-binding regulatory proteins that control the expression of genes and operons required for the utilization of specific carbohydrates [28]. Furthermore, the *ula*ABCDEF unit was found in all cases in spite of the observation of various species-specific gene rearrangements, insertions and losses (Figure 5). These modifications, together with sequence divergence between the orthologous pair genes, cause the exact nature of the metabolic pathway catalyzed by the homologous *ula*DEF units to become blurred with evolutionary divergence. The greatest conservation in operon structure and genomic context was found among the mammalian-associated Enterobacteria, both in the symbiotic intestinal microbiota as in human pathogens such as *Klebsiella pneumoniae *and *Salmonella enterica *serovar Typhi, with the *E. coli ula *regulon representing the reference sequence. Thus, the type *ula *regulon (Figure 5; Additional file 1) contains adjacent *ula*R and *ula*G genes and the *ula*ABCDEF operon, which is transcribed from the opposite strand. This cluster is flanked on its 5' end by hypothetical proteins of the CP4-57 prophage region and on its 3' end by genes encoding proteins of unknown function (e.g., DUF1471) and by the genes coding for ribosomal subunit proteins (e.g., *rps*F, for the 30S ribosomal subunit protein S6). In closely related Enterobacteria some of these contiguous genes are transcribed in the same direction as *ula*G or *ula*ABCDEF, with no intervening gene on the complementary strand, and they are conserved in multiple species (Figure 5, Additional file 1). These genes encode proteins with various functions but their physical proximity does not appear to bear any functional relevance to the utilization of L-ascorbate 6-phosphate. Among the enterobacterial genomes analyzed, only *Edwardsiella ictaluri *breaks this trend with its *ula *regulon flanked by genes encoding a hypothetical membrane protein and an *nfu*A-encoding Fe/S biogenesis protein.

A second group with a conserved operon structure includes genomes of Gram-negative *Proteobacteria *with diverse lifestyles, such as the environmental *Aeromonadales *bacterium *Tolumonas auensis*, the deep-sea *Photobacterium profundum*, the plant pathogen *Dickeya zeae *(the causative agent of soft rot in maize), and the human pathogens *Yersinia *spp., *Haemophilus *spp., and *Vibrio cholerae*. These microorganisms possess *ula *regulons that share various characteristics, such as a two-subunit PTS transporter wherein the large subunit (UlaAB)-encoding gene is equivalent to a gene fusion of *ula*A and *ula*B (Figure 5). In this group it is common to observe a gene rearrangement whereby the *ula*E or *ula*F genes (or both) are inserted between or next to the *ula*R and *ula*G cistrons (e.g., *H. ducreyi*); insertions of an unrelated gene encoding a Cof-like hydrolase are also common (e.g., *Y. intermedia*). Since these additional genes are to be co-transcribed from a different transcriptional unit than the remaining structural genes, their regulatory control might become less constrained and therefore freer to be fine-tuned in response to the metabolic pressures of the bacterium. Another shared feature is the completely unrelated genomic contexts among all these bacteria, although their *ula *regulons tend to be bracketed by genes encoding enzymes involved in sugar metabolism (e.g., transaldolases and transketolases in *T. auensis*, amylose in *V. cholerae*), redox cofactor biosynthesis (e.g., menaquinone and phylloquinone in *H. ducreyi*), and transcription factors (e.g., *D. zeae*, *V. cholera*).

In *Y. intermedia*, the *ula *regulon is flanked on either side by what appears to be N-terminally truncated genes corresponding to a transposase B (65 residues)/integrase (44 residues) enzyme pair; in *Y. enterocolitica*, the corresponding orthologs have been conserved in full length, and for example *Y. enterocolitica *integrase gene encodes a 450-amino-acid polypeptide that has 97% identity with the 44-residue (predicted) polypeptide in *Y. intermedia*. The flanking transposase/integrase cassette suggests that the *ula *regulon is part of a still mobile metabolic island (or islet) in *Y. enterocolitica *[4], whereas in *Y. intermedia *the mobility may have been abrogated by partial gene deletion. In addition, the finding of a syntenic association between the *ula *regulon and a nearby tRNA gene (yinte0001_t290) in *Y. intermedia *lends further support to the hypothesis that the *Y. intermedia ula *regulon belongs to an ancient mobile DNA segment [4].

While in Gram-negative bacteria the *ula*R/*ula*G cistrons are transcribed co-directionally, in all Gram-positive bacteria that we have analyzed one of the following applies: (1) *ula*R and *ula*G are transcribed divergently, with *ula*G being the first structural gene upstream of the PTS transporter genes (e.g., *Atopobium vaginae*, *Enterococcus faecium*, *Catonella morbi*, *Clostridium botulinum*); this is the most common scenario, and is similar in Gram-negative bacteria, with *ula*G-*ula*A intergenic distances from 250-500 bp; (2) *ula*R and *ula*G are co-transcribed along with the rest of structural genes (*Lactobacillus casei*); in this case, *ula*G-*ula*CAB intergenic distance shrinks to 5-45 bp, as is expected for genes co-transcribed in the same operon; or (3) *ula*R coding the DeoR-type regulator is absent, having been replaced by a *bgl*G gene encoding a 550-amino-acid residue BglG transcription factor containing two PRD (PTS regulatory domain) domains and an Arc repressor DNA-binding domain (*Streptococcus *spp.). In the latter case *bgl*G is always co-transcribed with *ula*G and comes immediately preceding (*S. pyogenes*) or following it (*S. pneumoniae*).

We also detected at least four duplicated DNA segments among the multi-species *ula *regulons examined (Figure 5). The first two cases (*P. profundum ula*R and a *C. morbi *gene coding for an uncharacterized hypothetical protein that is found after *ula*F) involve a single gene duplication event which, after divergence, retain about 60% sequence identity and are co-localized within the operon next to each other on the same strand, and therefore may both be functional in the context of L-ascorbate 6-phosphate degradation. A third case is found in *Y. intermedia*, where the *ula *regulon lacks a gene for UlaF; instead, two ORFs (yinte0001_31020 and yinte0001_18310) appear to have arisen by gene duplication from an ancestral *ula*F gene but these are located in genomic loci far apart from the *ula *regulon and from one another. Given that the rest of the operon has been conserved, it is plausible that the UlaF-like encoded proteins complement the function lost from the *ula *regulon. Finally, the most dramatic duplication event was observed in *S. termitidis*, where the entire regulon (except the gene for the repressor) seems to have undergone a duplication event, the result of which was two reversely transcribed copies. Despite being a Gram-negative bacterium, the *ula *regulon of *S. termitidis *lacks an *ula*R orthologous gene; instead, it contains a BglG-encoding gene, similar to that found in *Streptococcus *spp. Several gene insertions might have occurred since the whole-operon duplication event because the inserted genes were not affected by the duplication; inserted genes include those encoding a sulfatase, a Cof-like hydrolase, and several autotransporters.

In summary, the structure of the *ula *regulon has been considerably conserved across Gram-negative and Gram-positive bacteria despite various species-specific gene rearrangements, fusions, duplications, insertions, and deletions. Although it is not possible to establish beyond doubt that the specific functions of each of the genes in the *ula *regulon are strictly conserved throughout evolution, the conservation of sequence and operon structure suggests a common functional role for these genes in the catabolism of phosphorylated sugars, mainly L-ascorbate 6-phosphate, via the pentose phosphate pathway.

### Gene loss

In addition to gene duplication, the selective loss of genes in evolving lineages contributes to the diversification of gene inventories [29]. The role of gene loss in the molecular evolution of a protein family within a set of closely related genomes can be gauged by comparing the species tree with phylogenies constructed from available protein sequence data. If the existence of an ancestral gene and its vertical transmission to evolving lineages can be stated confidently, then the absence of the gene in a particular descendent lineage can be explained as a gene loss event. In this case, the gene tree will logically lack the particular branch in the species tree where gene loss has occurred. Based on well-documented reports [29], gene loss is generally attributed to negative selection processes that increase the genome's fitness by reducing its size and eliminating dispensable genes. In the case of *ula*GL, we first compared our protein tree (Figure 3) with published species trees of *Gammaproteobacteria *[22,23], which provided evidence for differential loss of *ula*GL across several genera. For example, in the enteric *Yersinia*, some species have lost *ula*GL (e.g., *Y. pestis*) while others (*Y. intermedia *and *Y. ruckeri*) have retained it. The same pattern of species-specific gene loss is apparent within most genera, like *Vibrio *and *Pasteurella*. An extreme case includes both highly specialized bacteria (like the endosymbionts *Buchnera *and *Wigglesworthia*) and more distantly related gammaproteobacterial genera (*Shewanella*, *Pseudomonas*, *Xylella*, and *Xanthomonas*), the genomes of which do not have a detectable *ula*GL. This absence may respond to either a lack of *ula*GL in the gammaproteobacterial ancestor, or to its presence but subsequent loss due to negative selection pressures.

With the possible exception of the *Firmicutes *(which are better represented in the UlaGL phylogeny), the presence of *ula*GL in a limited set of Gram-positive bacteria from diverse and distantly related phyla such as *Actinobacteria*, *Fusobacteria *and *Spirochaetes *likely reflects gene loss. Within the *Firmicutes*, the presence of UlaGL in several abundant genera (e.g., *Clostridium*, *Streptococcus*, *Lactobacillus*, and *Enterococcus*) has many exceptions at the species level that are best interpreted as gene loss events. For example, even though *C. butyricum*, *C. botulinum*, and *C. perfringens *all possess an *ula*GL gene, most other *Clostridium *spp. do not, including important human pathogens as *C. difficile*, *C. tetani *or *C. sordellii*. It therefore appears that *ula*GL has been retained only in particular Gram-positive genomes where it may contribute a fitness advantage.

In all cases analyzed (either *Proteobacteria *or Gram-positive bacteria), loss of *ula*GL was always associated with the complete loss of the *ula *regulon, indicating that the functions conferred by the *ula *genes are highly coordinated.

### Structural homology

**Figure 6:**
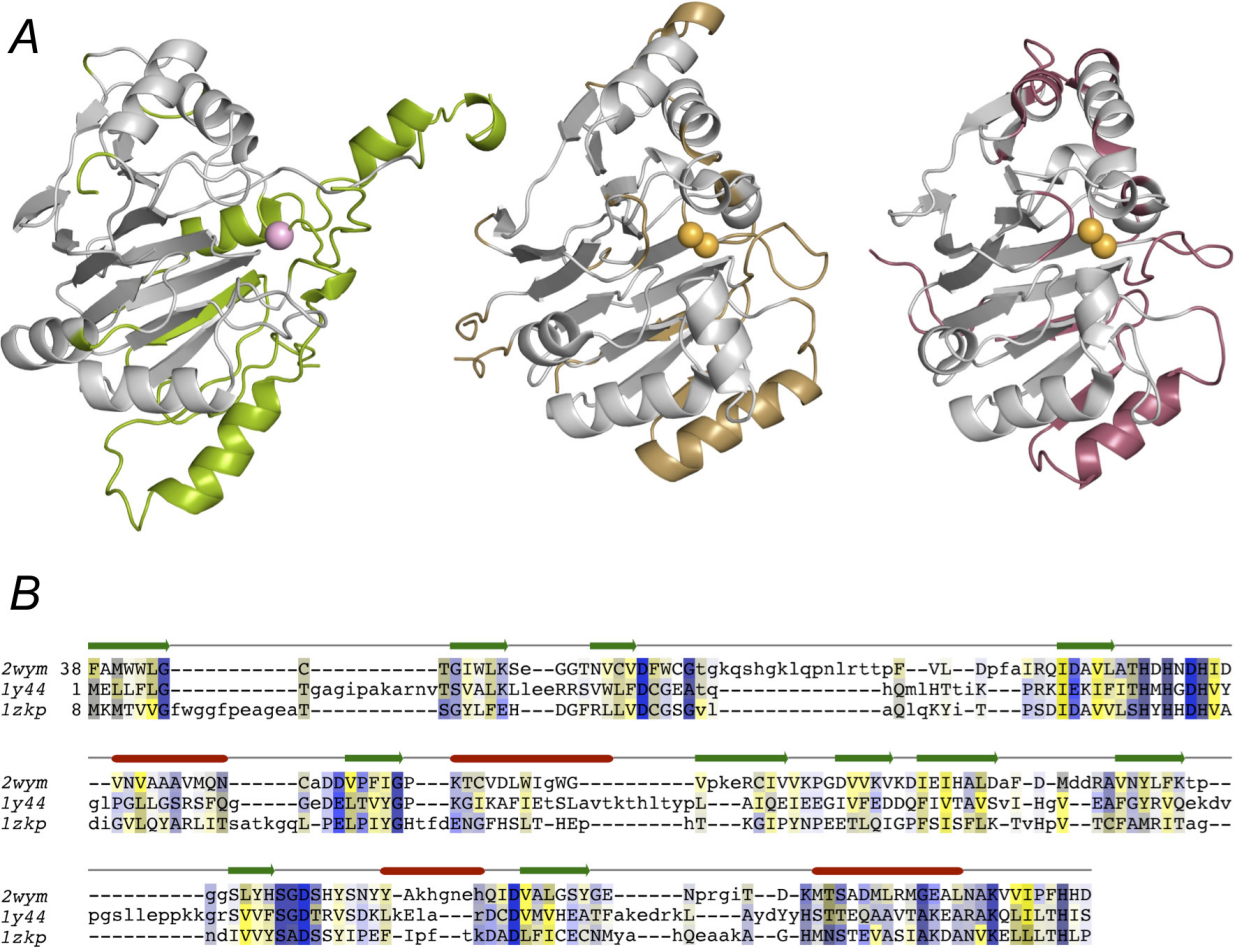
**Origin of UlaGL from an ancient MBL RNase sequence**. *A*. Structures of *E. coli *UlaG (PDB code 2wym, left), tRNA maturase RNase Z (PDB code 1y44, center) and *B. anthracis *ElaC homolog (PDB code 1zkp, right), with the conserved core domain shown in gray. Unique structural features are highlighted in green (UlaG), blue (1y44), and violet (1zkp). The Mn^2+ ^ion (violet) in UlaG and the Zn^2+ ^ions (orange) in the two RNases are shown as spheres. *B*. Multiple sequence alignment of UlaGL sequences (*E. coli *UlaG on top) and related RNase Z sequences obtained by structure-guided sequence searching and manual correction of the resultant alignments.

As we have previously discussed [18], the structure of *E. coli *UlaG strongly resembles RNA-processing enzymes of the MBL superfamily and yet their sequence identity is very low (14-18%) (Figure 6). Among these, the closest structural homologs found by SSM PDBeFold [30] include Zn^2+^-dependent RNases that catalyze phosphodiester cleavages in tRNA or mRNA substrates and recognize the three-dimensional structure of their RNA substrates. This group of enzymes includes the pre-mRNA 3'-end-processing endonuclease (PDB ID code 2i7t) [31], tRNA maturase RNase Z (PDB ID code 1y44) [32], and the ElaC homolog of *Bacillus anthracis*, a putative ribonuclease (PDB ID code 1zkp) (unpublished) (Figure 6). The number of aligned residues across all detectable structural homologs was 186 over 13 secondary structure elements, with an overall root mean-square deviation (r.m.s.d.) of 2.1 Å and an overall Q-score of 0.26. The pattern of conserved secondary structure elements consists of the β-sheet core (12 β-strands), except for the two last flanking β-strands (βN in *E. coli *UlaG) and the C-terminal helix (β14 in *E. coli *UlaG). Not unexpectedly, the hexamerization motifs in *E. coli *UlaG are missing from this conserved core. Further to the global similarity, the RNase Z enzymes appear to show strong similarity to UlaG at the level of the active site configuration and metal coordination sphere, thereby suggesting active-site conservation of the essential features that support hydrolytic activity in both enzyme classes. The similarities observed are remarkable since these RNases come from very broad phylogenetic origins, such as pre-mRNA 3'-end-processing endonuclease (human) and tRNA maturase RNase Z (bacteria). As noted above, the first iteration of PSI-BLAST with *E. coli *UlaG as the query had already identified with low scores (24% coverage, E-value 0.048, 35% identity over 90 residues) the putative mRNA 3'-end processing factor of the Crenarcheote *Pyrobaculum *spp. (*P. arsenicum *and *P. islandicum*), thus indicating a significant remaining sequence similarity despite enormous divergence.

To find more ancient relatives of UlaG among MBL RNases, we used a structure-guided sequence alignment to construct a profile Hidden Markov (HMM) model [33] to probe the complete non-redundant protein sequence database. Because of the current scarcity of UlaGL protein structures, the number of sequences present in the HMM alignment was necessarily low (6). The search with the HMM pattern retrieved 3929 sequences with a score better than 1 × 10^-10^, which included many sequences already identified by sequence-only searches as well as others from bacterial, archeal, and eukaryotic genomes. Apart from putative lactonases and hydrolases, the search found many sequences for RNase Z, YhfI, AtsA/ElaC, ATP-binding PhnP proteins, lipoate-protein ligase B, aryl- and glycolsulfatases, lipoyl/octanoyl transferases, cAMP phosphodiestarases, tRNA 3' processing enzymes, tRNA endonucleases, and the multidrug resistance protein RomA. More than 25% of the sequences were annotated as conserved hypothetical, predicted, or unnamed products, and many of the annotations were not curated (i.e. generated by automatic, database annotation). Despite the poor characterization of most of these sequences, it becomes evident that the descendants of the ancestral sequence from which modern *ula*G-like genes originated have a very broad phylogenomic distribution covering Bacteria, Archaea and Eukarya. The HMM searches suggest that the UlaGL sequences in eukaryotic organisms have roles mostly in RNA processing whereas in bacteria, a greater diversity of metabolic and functional roles is appreciable.

Furthermore, the recently determined structure of a MBL from *Brucella melitensis *subsp. abortus (PDB ID 3md7 and structures thereof) (unpublished) has revealed a monomeric enzyme with an Mn^2+^-dependent active site similar to UlaG and in contrast to the Zn^2+ ^ligand found in all other RNases. However, this enzyme represents a mixed case since it has only a marginally higher sequence identity with the above RNases (21%) than with UlaG (15%). Superposition of the two active sites (not shown) shows that the empty metal site in UlaG is occupied by a second Mn^2+ ^ion in the *B. melitensis *enzyme with a markedly different conformation of the liganded residues. In particular, the two bridging aspartates Asp90 and Asp188 (Asp121 and Asp226 in UlaG) adopt rotamers that bring the carboxylates closer to the Mn^2+ ^(site Zn2) ion by 0.6-1 Å, and two histidine residues in the coordination sphere of the second Mn^2+ ^in the *B. melitensis *enzyme (site Zn1) are either in a distinct conformation (His88, His119 in UlaG) or have no equivalent in UlaG (His170). The closest residue to His170 in UlaG is Asp184 (2.5 Å between Cα atoms), which is the last amino acid before an extensively disordered loop spanning residues 185-204. Only His228 in UlaG has no clear counterpart in the *B. melitensis *enzyme and could in principle play the role of His170; however, the 4.5-Å gap between the corresponding side chains renders this scenario unlikely. The *B. melitensis *structure was solved in the presence of various nucleoside monophosphates (GMP and AMP) that show a similar binding mode, characterized by the nucleotide phosphate buried deep within the metal center of the enzyme and establishing most of the stabilizing interactions, while the ribose moiety and, even less so, the guanine/adenine base, are more loosely bound. Given the chemical and structural similarities of L-ascorbate 6-phosphate with the ribose of GMP and AMP, and the fact that UlaG uses cyclic nucleotide analogs, we hypothesize that L-ascorbate 6-phosphate binds to UlaG in a similar orientation as GMP. This binding mode would position the substrate between the two long loops that are disordered in the UlaG structure, thus raising the possibility of an induced fit mechanism to occur upon substrate binding.

Taken together, structure-based sequence and HMM pattern searches and structure superpositions confirm the evolutionary relationship of the Mn^2+^-dependent UlaG family and an ancient family of Zn^2+^-dependent MBLs with RNase activity. Intriguingly, the recently solved structure of a second Mn^2+^-dependent MBL (from *B. melitensis*) lends further support to the notion that Mn^2+ ^was present in the ancestral RNase or in a wider branch of RNase/metabolic enzymes.

### Modeling UlaGL protein structures

**Figure 7:**
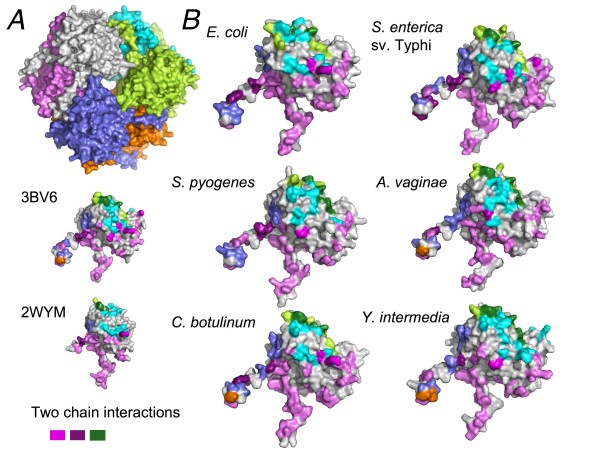
**Surface mapping of intersubunit contacts for modeled UlaGL structures**. *A*. Ribbon of an UlaGL hexameric structure in chain colors. *B*. Splayed-apart surface representation for a monomer of each modeled structure, shown with mapped intersubunit contacts in the same chain colors as in *A*.

Given the remarkable primary sequence conservation among many UlaGL proteins encoded by the human microbiome, including proteins from recognized human pathogens, we set out to model the hexameric structures of several representative sequences. The chosen set of protein sequences included *S. enterica *serovar Typhi, *C. botulinum*, *Y. intermedia*, *S. pyogenes*, and *A. vaginae*. All of these proteins share clear sequence patterns corresponding to the oligomerization motifs (Figures 1 and 2), suggesting that their structures will follow the hexameric architecture revealed by the two crystal structures available for UlaGL proteins [18]. Modeling was carried out with two well-established algorithms (see Methods section) and outcomes were inspected for consistency between the two methods. As controls, modeling of each original crystal structures (PDB ID 2wym and 3bv6) with one another converged to models that were essentially identical to the experimentally determined structure (within r.m.s.d. of 0.3 Å). Many sequence changes were conservative and did not generate geometric or electrostatic alterations in the modeled structures. Alterations of this nature might have indicated potential problems with the models. On the contrary, buried surface area (BSA) and other geometric and energetic statistics showed that the modeled structures were capable of forming native interaction surfaces. For example, the average BSA for the largest and second largest interface for the comparative models were, respectively, 4500 Å^2 ^and 1250 Å^2^, which compare well with the BSA for the same interfaces from the crystallographic full-length models (4000 Å^2 ^and 1350 Å^2^). Splayed-apart views of the contact surfaces for the modeled structures are shown in Figure 7 and further analyses are described in Additional files 13 and 14.

### Prediction of metal ligands

Prediction of the metal ligand for a particular MBL on the basis of primary sequence and structure alone is error-prone. This is because the same set of protein residues can bind more than one metal in similar or different coordination spheres, but also because the identity of metal ions that can bind into an active site of a particular protein depends, at least in part, on cellular regulatory mechanisms for metal ion homeostasis [34]. To date, four metal ions are known to bind to UlaGL enzymes, including Mn^2+^, Co^2+^, Zn^2+^, and Fe^3+^. For the first three (Mn^2+^, Co^2+^, and Zn^2+^) there is strong biochemical and biophysical experimental support [18]. However, the binding of Fe^3+ ^is so far backed only by its presence in an unpublished crystal structure (PDB ID 3bv6), and therefore its biochemical relevance will require a more extensive characterization. In *E. coli *UlaG, experimental evidence ranks Mn^2+ ^and Co^2+ ^as potential binders, since they display similar binding profile and both facilitate catalysis on L-ascorbate 6-phosphate, and singles out Zn^2+ ^as a potential competitive inhibitor of catalytic activity. Interestingly, under the cited experimental conditions *E. coli *UlaG does not bind Fe^3+ ^[18]. Collectively, this indicates that the UlaGL family proteins may be able to charge their active site with a broad selection of divalent and trivalent metal ions, thereby suggesting that these perform both carboxyl and phosphoryl ester hydrolysis as well as redox reactions. This versatility in metal selection fits nicely with the expected adaptability of the MBL scaffold fold to catalytically transform a wide range of biologically relevant nutrient molecules.

## Discussion

The recent determination of the X-ray crystal structure of *E. coli *UlaG, the first enzyme of the anaerobic catabolic pathway of L-ascorbate in Enterobacteria, has brought to light a novel fold type of the MBL superfamily [18]. This sequence and its associated structural features defines a new UlaGL family in the Uniprot database (http://www.uniprot.org) [35]. Much remains unknown about its phylogenetic origin, its evolutionary relatedness to RNA processing enzymes, and the potential plasticity of the structural elements that configure UlaG. In order to increase our understanding of the UlaG family we sought to study its phylogenetics and the consequences of the colocalization of the corresponding gene with genes coding proteins associated with the *ula *regulon. Here, we have retrieved and analyzed a set of near 200 bacterial and archeal sequences with detectable homology to UlaG and that we have denominated the UlaGL family.

A first set of closely homologous sequences prevails in related Gram-negative bacteria from the *Gammaproteobacteria *that include species from the orders *Enterobacteriales*, *Vibrionales *and *Pasteurellales*. Many, if not all, of these sequences come from bacteria from the mammalian microbiome and also from human, animal, and plant pathogens. Given their relatively high amino acid sequence identity (> 60%) and the likely presence of a shared genomic context homologous to genes in the *ula *regulon, the UlaG homologs constitute plausible orthologous sequences. Even relatively distant bacteria such as *V. cholerae*, an obligate anaerobe and human pathogen, conserves the *ula *regulon structure and has sequence identity to *E. coli *UlaG above 65% (Figure 2, Additional file 3), in addition to sharing the same structural scaffold with an r.m.s.d. of 1.5 Å for nearly the entire length of the two proteins. This strong pattern of conservation of the sequence and structure of both the structural genes and the regulatory sequences of the *ula *regulon across *Gammaproteobacteria *is in agreement with the evolution of Enterobacteria, and indicates that these sequences are of ancient origin [36-38] and under strong purifying (negative) selection [3]. Assuming vertical transmission of *ula*GL among the *Gammaproteobacteria*, the lack of *ula*GL along certain genera and species (e.g. *Y. pestis *and *C. tetani *lack the *ula *regulon) indicates a selective loss of the *ula*GL gene present in the ancestor to those lineages, or absence in the ancestor followed by acquisition from a distant source in several lineages. Given that HGT between closely related species is rare [29] and the congruence of the gene tree with the species tree for gammaproteobacterial genomes, the first scenario, involving gene loss of an ancestral gene, appears more likely to have played a significant role in shaping the phylogenomics distribution of the UlaGL family.

A second set of homologous UlaGL sequences are found in Gram-positive bacteria (mostly *Firmicutes *and *Actinobacteria*), again mainly among symbiotic and pathogenic mammalian-associated bacteria. A syntenic association between *ula*GL genes and an adjacent *ula*R-like gene is also strongly conserved in Gram-positive bacterial genomes, although in *Streptococcus *spp. the *ula*R-encoding DeoR repressor appears to have been substituted by a gene coding a more complex BglG-family transcription factor, which has two PRD (PTS regulatory domains) and an Arc DNA-binding domain. While the defining features of the *ula *regulon are conserved among Gram-negative and Gram-positive bacteria, there are numerous species-specific gene rearrangements and insertions, and even whole operon duplications, which might play adaptive roles in the particular lifestyle and metabolic requirements of each species. Thus, the plasticity of the *ula *regulon structure is in agreement with previous reports that suggest that gene sequences are generally more conserved than the gene organization within an operon, with the possible exception of genes encoding physically associated polypeptide chains, which tend to have a higher propensity to remain clustered [22].

The conservation of genomic context about the putative *ula*GL regulons/operons within closely related species indicates a similar functionality in carbohydrate/vitamin metabolism. Indeed, the presence of sequences of prophage origin flanking the *ula *regulon in several enterobacterial species and the flanking transposase/integrase genes found in *Yersinia *spp. are a strong indication of the coherent function as well as the potential for the lateral transfer of the entire regulon, and are consistent with the HGT events from an enterobacterial donor to Gram-positive recipients apparent from the reconstructed molecular evolution of *ula*GL (Figure 3). When larger evolutionary distances are considered, the genomic context becomes far less conserved. For example, for more distantly related Gram-negative species such as *Escherichia *spp. and *Yersinia *spp. the genome location of the *ula *regulons is unrelated.

Most interestingly, the combination of structural and sequence homology searches help delineate a complete phylogenetic tree of UlaG-related sequences with a broader scope than any individual approach might achieve. A first feature of such a tree is a core of bacterial sequences that share a closely related functionality in L-ascorbate 6-phosphate (or a similar metabolite) metabolism and which span Gram-negative and Gram-positive bacteria with diverse lifestyles (commensal, symbiotic, pathogenic, or free living). Although true orthology is a plausible scenario between UlaGL sequences with > 55% sequence identity with the *E. coli *reference sequence, at this stage we cannot exclude that in some organisms the *ula *operon was dedicated to the catabolism of other, related metabolites, such as a different hexose phosphate. Subtle changes in the active site might change the identity of the bound metal or the catalytic side chains, thereby modifying substrate specificity and/or reaction selectivity. Furthermore, the environment of the microorganism and its metal homeostasis [34] constrains the possible substrates, in addition to creating selection pressures in favor of particular metabolic outcomes. Therefore, the question of the specific function, among the relatively narrow set of choices compatible with the phylogenetic analysis, of the *ula *operon in each organism should be addressed in a species-specific fashion.

The combination of sequence searches with structural information has allowed the restoration of the distant evolutionary history of UlaGL proteins, whereby the most prominent feature is the relatedness of modern metabolic UlaGL enzymes with the widespread RNase Z essential enzyme. This relation strongly suggests that the forerunner to the modern UlaGL sequences was an RNase that was present in the last common ancestor of Eubacteria, Archea, and Eukarya. Key differences between these two protein families are the acquisition by UlaGL of N- and/or C-terminal sequences governing their quaternary architectures and the presence of multiple insertions/deletions in the loops that extend over the active site. The abstraction of the conserved sequence-structure features between UlaGL and RNase Z permits the reconstruction of even more distant (diverged) phylogenetic relationships of UlaGL with Zn^2+^-dependent hydrolases and oxidorreductases. The latter enzymes have previously been noted, on the basis of strict structure conservation, to form an independent lineage loosely termed class B3 β-lactamases and distinct from class B1+B2 β-lactamases [14]. Although structure-based profiles and sequence alignments were required to reliably establish the evolutionary relatedness between UlaGL and RNase Z, it is interesting to consider that the accumulation of high-quality sequencing data for many different species made it possible to retrieve at least one RNase Z sequence on iteration 1 (and later iterations) from sequence-only searches of *E. coli *UlaG using PSI-BLAST. This sequence is from the Crenarcheota *Pyrobaculum *spp., with E-score of 1 × 10^-4^, 24% sequence identity and 40% sequence similarity (over 201 amino-acid residues). Another putative homologous sequence that appears in PSI-BLAST searches but whose reliability has not been assessed owing to a lack of a crystal structure is the membrane protein RomA, which participates in multi-drug efflux. Interestingly, RomA from diverse pathogenic bacteria appears repeatedly in PSI-BLAST searches with scores that are marginally better to those of *Pyrobaculum *spp. RNase Z (E-score 7 × 10^-4^, 25% sequence identity, 41% sequence similarity, over 269 amino-acid residues). Finally, direct structure comparison will be required to establish that UlaGL and RomA transporters share a common ancestor and to rule out other possible scenarios for the sequence similarity observed.

Some structural features of UlaG stand in stark contrast with the overall fold plan of MBL RNA-processing enzymes, including the oligomerization motifs and the presence and location of putative substrate-binding elements. This distant homology detected only at the structural level allows us to put forward a hypothesis whereby the ancient fold type of MBLs, possibly devoted to an essential function in relation to RNA processing, has been duplicated and modified to accommodate new substrate specificities and new reactivities. Indeed, UlaG still retains measurable phosphodiesterase activity toward cyclic nucleotides as a side reaction [18].

Our phylogenetic analysis of the UlaGL family has allowed the definition of the structural and sequence features constituting the UlaGL fold and that differentiate it from other related MBLs. Since these features are derived in part from the available crystal structures and in part from systematic sequence searches, they have predictive power for comparative modeling for sequences from unknown structure. Indeed, most UlaGL close homologs can be confidently modeled (Figure 7). Our modeling exercise has confirmed that the distinctive hexameric complex is well conserved. The buried surface areas, surface complementary, and number of interface interactions are no less indicative of a strong interaction than for the two reference structures. Further details on the modeled sequences and evidence that the structures could exist as hexameric complexes are given in Additional files 13 and 14. No attempt was made to model sequences that did not have identities above 50% and for which the similarity did not extend along the complete length of the protein sequence.

The structure-guided phylogenetic analysis that we have performed shows that the diversity of sequences related evolutionarily to UlaG is much greater than previously anticipated. UlaGLs are present in several bacterial lineages, from mammalian symbionts to free-living marine bacteria, and are found even among archeal and eukaryotic sequences. By combining available crystal structures with molecular evolution methods we have provided solid support to the idea that the modern UlaGL family of carbohydrate and vitamin metabolism originated from an ancient RNA processing enzyme by the addition of N and C-terminal oligomerization motifs and the specialization of its active site. The spread of UlaGL (along the *ula *regulon) among symbiotic and pathogenic bacteria by HGT, which is supported by our Bayesian and maximum likelihood tree reconstructions, suggests that UlaGL may have conferred a growth advantage under some environmental and community pressures. Finally, the analysis presented here, combined with future ones gathered from all sequence and structure data that will be generated, will afford a better understanding of the UlaGL family and, by extension, of other subfamilies of the wide MBL trunk of sequence space.

## Conclusions

The emergence of new functions from pre-existing folds by gene duplication and divergence of the new copy represents an enormous source of genetic variation in prokaryotic genomes, which is further expanded by HGT and genomic rearrangement events. The gene encoding UlaG in commensal and symbiotic enterobacteria of the human gastrointestinal tract is an example of the result of an ancient gene duplication event from a gene which may have had an essential RNA metabolizing function in the last common ancestor of Bacteria, Archaea, and Eukarya. Our phylogenetic analyses, based both on sequence-only and structure-guided multiple sequence alignments, provide solid support for this hypothesis, and reveal a greater phylogenetic distribution for genes encoding UlaGL than previously anticipated, including the possibility of multiple independent gene duplications along bacterial and archeal lineages, as well as of lineage-specific gene loss. On the basis of these analyses, we propose that the ancestral RNase-encoding gene adopted its new metabolic role in the context of L-ascorbate metabolism by grafting both N and C-terminal motifs onto the RNase scaffold while extensively modifying the active-site loops responsible for binding the metal and substrate(s) and exerting the catalytic function(s). This scenario, i.e. the creation of new functions by modification of the extremes and critical functional loops in the core of a pre-existing MBL domain, as well as by selection of a more suitable metal ion (an aspect frequently overlooked in MBL enzymes in favor of an assumed Zn^2+ ^metal center), might provide clues as to the parts of an MBL protein sequence that can accommodate most variation and therefore represent important determinants of, for example, the generation of new antibiotic resistance mechanisms.

## Methods

### Generation of the UlaGL sequence data sets

In order to restrict the initial phylogenetic analysis to the set of core UlaG orthologs, we searched the NCBI nr database with BLASTP [39] applying an E-value < 1 × 10^-10 ^and a match length > 75% of the query length (265 amino acids). Of the 220 sequences found to fulfill the E-value criterion, 91% or 201 sequences had an aligned length greater than 265 residues. This last criterion proved crucial to prune sequences that retained homology to the core MBL domain while having lost several UlaG characteristic features, such as the 20-amino-acid residue N-terminal and the C-terminal oligomerization motifs. The 201 single copy genes that met these homology criteria were further trimmed on the basis of sequence identity (sequences with > 50% sequence identity were retained) and pruned from duplicated sequences; the resultant group, containing 175 sequences, was used for phylogenetic reconstruction. Concatenated sequences of these 175 genes were aligned using ClustalX v. 10.0.2 [40] or Muscle v. 3.6 [41] and Gblocks v. 0.91b [42] was applied to edit the alignment to remove gaps and poorly aligned regions. The final alignment contained 333 aligned amino acid characters of the original 415 (80%).

We then sought to find more distantly related UlaGL sequences by searching the NCBI protein and nucleotide sequence databases for fully sequenced genomes (December 2008) using either PSI-BLAST (BLASTP v. 2.2.24+, non-redundant protein databases, 1000 maximum target sequences, profile-inclusion threshold of expected E-value 0.005, four iterations) or tBLASTn (translated nucleotide databases) from the NCBI Blast2 interface (http://blast.ncbi.nlm.nih.gov/Blast.cgi) [39]. After the initial sequence collection, the full NCBI database was probed with individual UlaG homologous sequences to enhance the chances of Finding sequences related to particularly divergent MBLs and to better define the range of organisms containing these proteins. Sequences were aligned with ClustalX or Muscle with default alignment parameters and unnecessary gaps and poorly aligned regions were pruned with Gblocks. The subset of archeal UlaGL homologs were selected from the original multi-species alignment, re-aligned to emphasize archeal-specific sequence features, and prune with Gblocks for phylogenetic purposes. The archeal UlaGL alignment had 264 aligned amino acid characters from 17 taxa.

To retrieve even more distant UlaGL sequences, we first constructed a structure-based sequence alignment using the superimposed homologous structures identified with PDBeFold [30] (see below) and used this alignment, with 5 sequences and 232 aligned amino acid residues, as seed for the calculation with HMMER v. 3.0 (http://hmmer.org/) of a profile hidden Markov model (HMM) [33]. This HMM profile was then queried against the NCBI nr database with default parameters to retrieve sequences with significant similarity to the profile HMM. For a representative subset of 30 RNase Z sequences identified by HMMER with high E-values (< 1^-10^) we built a pruned multiple sequence alignment as described above (Figure 6).

A complete listing of bacterial sequences, taxonomy, accession numbers and their sequence identities with respect to *E. coli *UlaGL is provided as Additional file 3. The complete sequence sets and corresponding alignments are provided as Additional files 4, 5 and 6, and 9, 10 and 11.

### Phylogenetic tree generation

**Figure 8:**
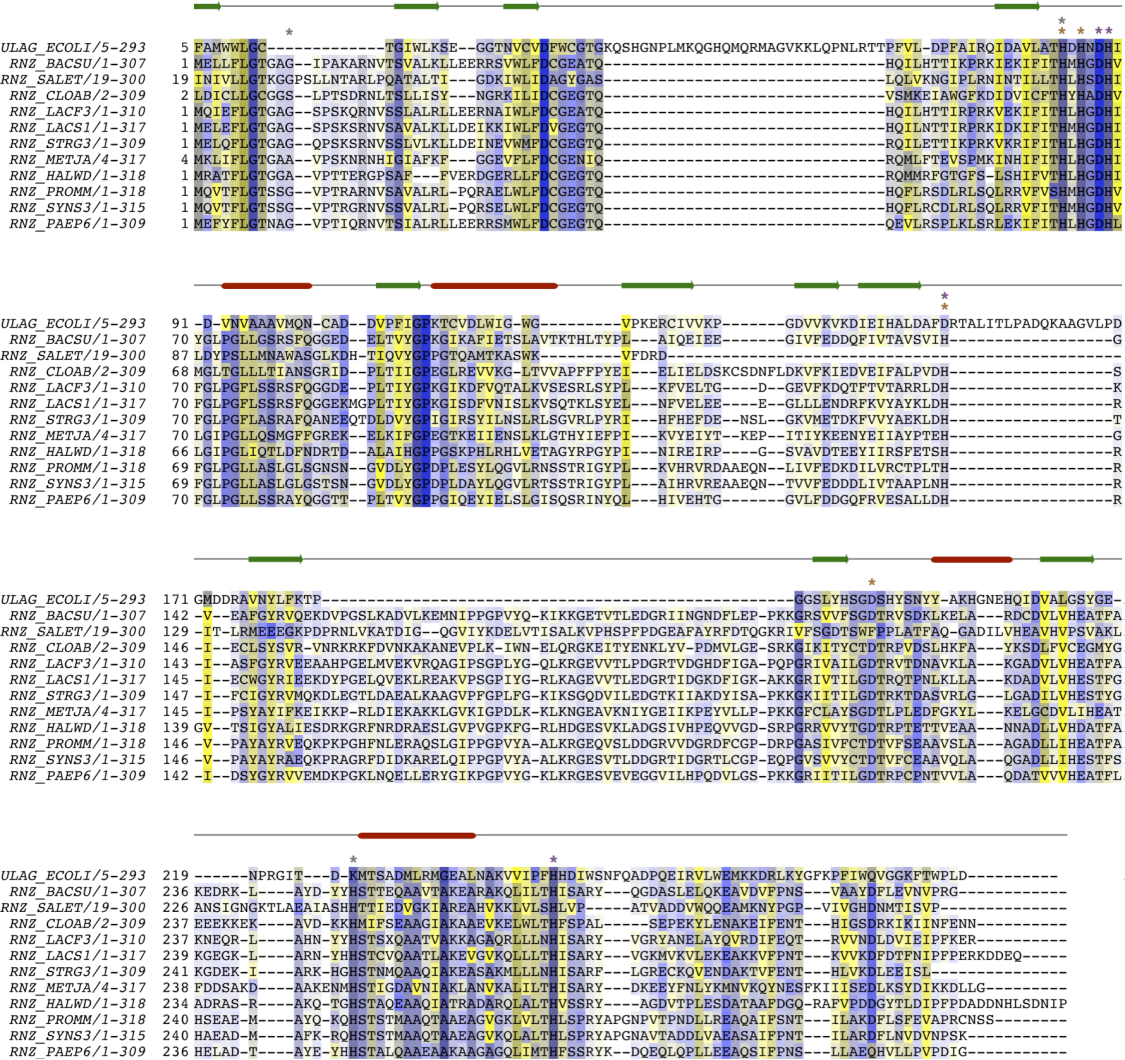
**Sequence alignment of UlaGL/RNase Z homologs**. Multiple sequence alignment of *E. coli *UlaG and homologous RNase Z sequences from selected Bacteria and Archea, with secondary structure overlaid above. Residues in contact with Mn^2+ ^in *E. coli *UlaG are indicated with gray asterisks, and key histidine and aspartate residues in MBL conserved motifs I-V are marked with orange and purple asterisks, respectively.

Maximum likelihood (ML) and Bayesian Inference (BI) phylogenetic analysis were carried out with RAxML v. 7.0.4. [43] and MrBayes v. 3.1.2 [44], respectively, using the concatenated multi-species alignment files for the complete UlaGL bacterial orthologous sequences (Figure 3), the archeal homologous sequences (Figure 5), and the distant MBL RNase Z alignment (Figure 8). RAxML was run with the PROTCATWAG model, with 100 bootstrap replicates. Bayesian consensus trees were generated with MrBayes using amino acid substitution rates and state frequencies fixed to the WAG parameters [45]. A uniform (0.0, 200.0) prior was assumed for the shape parameter of the gamma distribution of substitution rates [46], an unconstrained exponential prior with rate 10.0 for branch lengths, and all labeled topologies were a priori equally probable. Four independent MCMC analyses were run, each with one cold chain and three heated chains, with the incremental heating schema implemented in MrBayes (λ= 0.2). Convergence was assumed after the topology of the samples from the two cold chains had reached an average standard deviation of split frequencies (SDSF) of less than 0.01 (after 5,032,000 generations). The resulting trees were drawn with FigTree (http://tree.bio.ed.ac.uk/software/figtree/). No significant changes in the topology of the trees were observed when comparing the RaxML and MrBayes results, although differences in statistically unsupported regions of the trees were common.

In addition to the complete bacterial UlaGL tree, a tree containing a set of gammaproteobacterial species was generated with Tree-Puzzle v. 5.2 [47] using established methods [22,23]. The congruence between the UlaGL tree and the species tree was assessed by the Shimodaira-Hasegawa (SH) [48] test as implemented in the same software package.

All phylogenetic trees are provided as Additional files 7, 8 and 12.

### Genomic context

Genomic context was retrieved from the Entrez database [49], where records were available, or via the CDS records with the NCBI genome entry. Transcription orientation was consulted with EnsemblBacteria (http://bacteria.ensembl.org/) or the Genome Reviews tool (http://www.genomereviews.ebi.ac.uk/).

### Structural comparisons and modeling

Structural homologs of UlaG were found with PDBeFold [30] using the crystal structures of apo- and Mn^2+^-loaded *E. coli *UlaG (PDB ID 2wyl and 2wym, respectively) [18] with default cut-off parameters (70% identity of secondary structure in the target and the query) as search probes. Modeling of homologous hexameric UlaGL structures were performed with MODELLER-9v8 [50] and Rosetta v. 3.2 [51,52] using customized python scripts and pair-wise sequence alignments extracted from the multiple sequence alignments used for phylogenetic analyses (see above). In MODELLER-9v8, 100 models were generated using the standard 'automodel' with additional symmetry restraints to keep similar conformations for each chain of the hexamer. The models were assessed using Z-DOPE, a normalized atomic distance-dependent statistical potential based on known protein structures. The Z-DOPE scores ranged from -1.11 to -1.62, where a score less than -1 indicates a 'reliable' model (i.e., 80% of its Cα atoms are within 3.5 Å of their correct positions). Hexameric models with the best Z-DOPE scores were subjected to loop refinement of the residues involved in the oligomerization motifs (Figure 1). For each loop, 50 models were generated using the 'loop' routine of MODELLER (with additional symmetry restraints, as above). The final loop conformations were then selected based on their Z-DOPE scores. Finally, the best models were subjected to side chain refinement using SCWRL4 [53]. Although some post side chain refinement models had worse Z-DOPE scores than unrefined models, they exhibited better stereochemistry (Additional file 13) and are therefore likely to be more accurate than the unrefined models. The Z-DOPE scores for the final refined hexameric models ranged from -1.20 to -1.68. Not surprisingly, the control hexameric crystal structures (PDB codes 2wym and 3bv6) received the best scores (-2.10 and -1.84, respectively). Stereochemistry was assessed using MolProbity [54]. In addition, for each hexamer model, the interfaces were evaluated with ModTie, a residue contact statistical potential derived from binary domain interfaces in known structures (Additional file 13) [55]. Interface Z scores that are less than -2 indicate a 'possible' surface. The interfaces of all hexamers scored lower than -2, and the scores were not appreciably correlated with the percent identity to the template structures. The analysis of surface area and shape complementarity (Additional file 14), as well as all figures showing structures and superpositions, were carried out with PISA [56] (http://www.ebi.ac.uk/msd-srv/prot_int/pistart.html) and PyMOL (http://www.pymol.org/).

## List of abbreviations

(BSA): buried surface area; (GIT): gastrointestinal tract; (HGT): horizontal gene transfer; (HMM): Hidden Markov model; (MBL): Metallo-beta-lactamase; (r.m.s.d.): root mean-square deviation; (TIM): Triose phosphate isomerase; (*ula*): utilization of L-ascorbate; (UlaGL): UlaG-like.

## Authors' contributions

FJF performed most structural and phylogenetic analyses and edited the manuscript. FG and MLE participated in the structural analysis. JA, LB, MC and JB edited the manuscript. MCV conceived and designed the overall study, supervised all analyses and wrote the manuscript. All authors read and approved the final manuscript.

## Supplementary Material

Additional file 1 **Figure S1**. Genetic organization and transcriptional direction of the utilization of L-ascorbate (*ula*) regulon in *Escherichia coli *and encoded structural genes. Click here for file

Additional file 2 **Figure S2**. Catabolism of L-ascorbate 6-phosphate in enterobacteria and established catalytic activity of *E. coli *UlaG. Click here for file

Additional file 3 **Table S1**. Sequences included in the phylogenetic analyses. Click here for file

Additional file 4 **Bacterial UlaGL sequences**. FASTA file containing the complete set of bacterial UlaGL sequences. Click here for file

Additional file 5 **Filtered multiple sequence alignment of bacterial UlaGL sequences**. Multiple sequence alignment of bacterial UlaGL sequences in FASTA format prior to Gblocks filtering. Click here for file

Additional file 6 **Unfiltered multiple sequence alignment of bacterial UlaGL sequences**. Multiple sequence alignment of bacterial UlaGL sequences in FASTA format after Gblocks filtering, used for phylogenetic reconstruction. Click here for file

Additional file 7 **Phylogenetic tree of bacterial UlaGL**. Majority rule consensus tree of bacterial UlaGL sequences calculated with MrBayes and RAxML (used to prepare Figure 3). Nodes are labeled with BIPP (Bayesian inference posterior probability) and MLBP (maximum-likelihood bootstrap percentage). Click here for file

Additional file 8 **Gammaproteobacterial species tree used for comparison with UlaGL tree**. Phylogenetic tree constructed from a set of representative *Gammaproteobacteria *species that encode UlaGL, used for the evaluation of potential HGT events between related gammaproteobacterial species. Internal nodes are labeled with maximum-likelihood branch length support (MLBL). Tree was calculated with Tree-Puzzle v. 5.2 Click here for file

Additional file 9 **Archeal UlaGL sequences**. FASTA file containing the set of archeal UlaGL sequences. Click here for file

Additional file 10 **Unfiltered multiple sequence alignment of archeal UlaGL sequences**. Multiple sequence alignment of archeal UlaGL sequences in FASTA format prior to Gblocks filtering. Click here for file

Additional file 11 **Filtered multiple sequence alignment of archeal UlaGL sequences**. Multiple sequence alignment of archeal UlaGL sequences in FASTA format after Gblocks filtering, used for phylogenetic reconstruction. Click here for file

Additional file 12 **Phylogenetic tree of archeal UlaGL**. Majority rule consensus tree of archeal UlaGL sequences (using two eubacterial sequences as outgroups) calculated with MrBayes and RAxML (used to prepare Figure 5). Nodes are labeled with BIPP (Bayesian inference posterior probability) and MLBP (maximum-likelihood bootstrap percentage). Click here for file

Additional file 13 **Table S2**. Homology modeling, interfaces, and geometry validation of UlaGL homology models generated with MODELLER-9v8. Click here for file

Additional file 14 **Table S3**. Analysis of buried contact area by PISA of reference structures and homology models generated with MODELLER-9v8. Click here for file
