# Supplementary figures and images for: The UlaG protein family defines novel structural and functional motifs grafted on an ancient RNase fold
Source: BMC Evol Biol. 2011 Sep 26;11:273. doi: 10.1186/1471-2148-11-273 (PMC3219644; doi:10.1186/1471-2148-11-273)

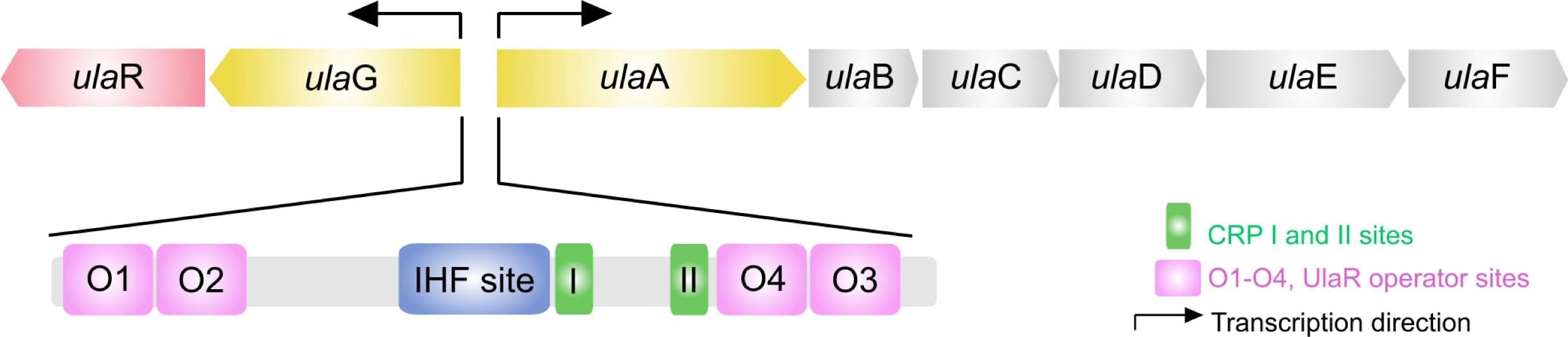

Supplement: Additional file 1 — Figure S1. Genetic organization and transcriptional direction of the utilization of L-ascorbate (ula) regulon in Escherichia coli and encoded structural genes. [file 1471-2148-11-273-S1.JPEG]

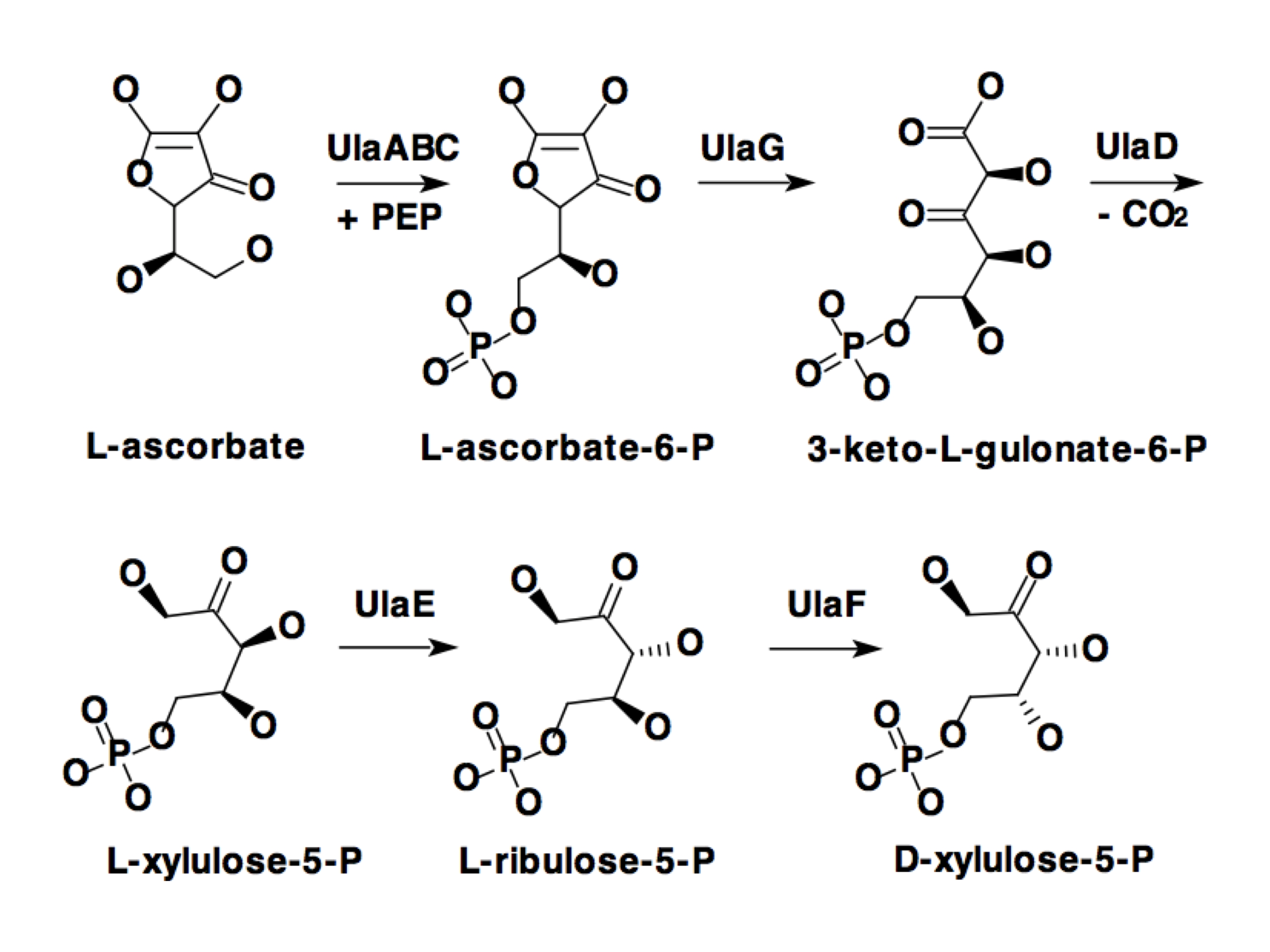

Supplement: Additional file 2 — Figure S2. Catabolism of L-ascorbate 6-phosphate in enterobacteria and established catalytic activity of E. coli UlaG. [file 1471-2148-11-273-S2.JPEG]
