# Supplementary material for: The UlaG protein family defines novel structural and functional motifs grafted on an ancient RNase fold
Source: BMC Evol Biol. 2011 Sep 26;11:273. doi: 10.1186/1471-2148-11-273 (PMC3219644; doi:10.1186/1471-2148-11-273)
Supplement: Additional file 3 — Table S1. Sequences included in the phylogenetic analyses. [file 1471-2148-11-273-S3.PDF]

Additional File 3 -- Table of all UlaGL orthologous protein sequences used for phylogenetic tree reconstruction.

GI code	Genus	Species	Strain	% Id.	Comments
**Proteobacteria > Gammaproteobacteria > Enterobacteriales > Enterobacteriaceae**					
110644549	Escherichia	coli	536	99	Human uropathogenic (UPEC)
188495101	Escherichia	coli	53638	99	Human enteroinvasive (EIEC)
194439543	Escherichia	coli	101-1	99	Human enteroaggregative (EAEC)
26251087	Escherichia	coli	CFT073	99	Human uropathogenic (UPEC)
260450981	Escherichia	coli	DH1	99	Laboratory strain
193066001	Escherichia	coli	E22	99	Human enteropathogenic (EPEC)
307558360	Escherichia	coli	H299	99	Human commensal
307146934	Escherichia	coli	M605	99	Mammal commensal
300940679	Escherichia	coli	MS 21-1	99	Human commensal
301325956	Escherichia	coli	MS 78-1	99	Human commensal
15804781	Escherichia	coli	O157:H7 EDL933	100	Human enterohaemorrhagic (EHEC)
261225312	Escherichia	coli	O157:H7 str. FRIK2000	100	Bovine isolate
261255436	Escherichia	coli	O157:H7 str. FRIK966	100	Bovine isolate
218561351	Escherichia	coli	S88	99	Human meningitis isolate
170682852	Escherichia	coli	SMS-3-5	99	Highly antibiotic resistance strain
89110912	Escherichia	coli	str. K-12 substr. W3110	100	Laboratory strain
170766945	Escherichia	albertii	TW07627	99	Human candidate enteropathogenic
307228898	Escherichia	coli	TA206	99	Mammal commensal
218551462	Escherichia	fergusonii	ATCC 35469	99	Mammal commensal
254037212	Escherichia	sp	1_1_43	99	Human commensal
187730696	Shigella	boydii	CDC 3083-94	99	Human enteroinvasive, dysentery
91207971	Shigella	boydii	Sb227	99	Human enteroinvasive, dysentery
82779392	Shigella	dysenteriae	Sd197	99	Human enteroinvasive, dysentery
24115547	Shigella	flexneri	2a str. 301	99	Human enteroinvasive, dysentery
110808107	Shigella	flexneri	5 str. 8401	99	Human enteroinvasive, dysentery
91207973	Shigella	flexneri		99	Human enteroinvasive, dysentery
157147841	Citrobacter	koseri	ATCC BAA-895	99	Human neonatal sepsis and meningitis
283786865	Citrobacter	rodentium	ICC168	95	Mouse pathogen
237729120	Citrobacter	sp	30_2	99	Human commensal
291085977	Citrobacter	youngae	ATCC 29220	98	Presumptively pathogenic
261342849	Enterobacter	cancerogenus	ATCC 35316	96	Rarely pathogenic in humans
311281262	Enterobacter	cloacae	SCF1	95	Nosocomial pathogen
296100955	Enterobacter	cloacae	subsp. cloacae ATCC 13047	95	Nosocomial pathogen
295098339	Enterobacter	cloacae	subsp. cloacae NCTC 9394	95	Nosocomial pathogen
206578410	Klebsiella	pneumoniae	342	96	Opportunistic human pathogen
152973057	Klebsiella	pneumoniae	subsp. pneumoniae MGH 78578	96	Opportunistic human pathogen
288937514	Klebsiella	variicola	At-22	96	Soil and plant reservoirs
161505118	Salmonella	enterica	subsp. arizonae serovar 62:z4,z23:-- str. RSK2980	97	Human pathogen
162139503	Salmonella	enterica	subsp. enterica serovar Choleraesuis str. SC-B67	97	Swine pathogen
205355137	Salmonella	enterica	subsp. enterica serovar Gallinarum str. 287/91	97	Fowl typhoid
168245170	Salmonella	enterica	subsp. enterica serovar Heidelberg str. SL486	97	Human pathogenic
224586221	Salmonella	enterica	subsp. enterica serovar Paratyphi C strain RKS4594	97	Typhoid fever
16763199	Salmonella	enterica	subsp. enterica serovar Typhi str. CT18	98	Typhoid fever
213612489	Salmonella	enterica	subsp. enterica serovar Typhi str. E98-2068	97	Typhoid fever
242237579	Dickeya	dadantii	Ech703	74	Plant pathogen
251787936	Dickeya	zeae	Ech1591	74	Plant pathogen
238921505	Edwardsiella	ictaluri	93-146	82	Channel catfish pathogen
269140631	Edwardsiella	tarda	EIB202	83	Freshwater animals/human pathogen
238793216	Yersinia	intermedia	ATCC 29909	74	Rarely pathogenic in humans
238753547	Yersinia	ruckeri	ATCC 29473	70	Fish pathogen
212712639	Providencia	alcalifaciens	DSM 30120	69	Human enteropathogen
268593234	Providencia	rettgeri	DSM 1131	71	Human enteropathogen
261346715	Providencia	rustigianii	DSM 4541	69	Human enteropathogen
**Proteobacteria > Gammaproteobacteria > Pasteurellales > Pasteurellaceae**					
33152858	Haemophilus	ducreyi	35000HP	65	Human pathogen
167855699	Haemophilus	parasuis	29755	63	Swine pathogen
254363139	Mannheimia	haemolytica	PHL213	63	Bovine pneumoniae
261492984	Mannheimia	haemolytica	serotype A2 str. BOVINE	63	Bovine pneumoniae
161511013	Mannheimia	succiniciproducens	MBEL55E	66	Bovine commensal
260912890	Pasteurella	dagmatis	ATCC 43325	63	Canine/feline pathogen
15602628	Pasteurella	multocida	subsp. multocida str. Pm70	62	Animal/human pathogen
257465367	Actinobacillus	minor	202	63	Animal pathogen
240949865	Actinobacillus	minor	NM305	63	Animal pathogen
307260061	Actinobacillus	pleuropneumoniae	serovar 11 str. 56153	62	Swine pathogen
165977136	Actinobacillus	pleuropneumoniae	serovar 3 str. JL03	62	Swine pathogen
126209165	Actinobacillus	pleuropneumoniae	serovar 5b str. L20	63	Swine pathogen
152977923	Actinobacillus	succinogenes	130Z	66	Nonpathogenic industrial microorganism
261867733	Aggregatibacter	actinomycetemcomitans	D11S-1	62	Periodontopathogen
251792967	Aggregatibacter	aphrophilus	NJ8700	62	Human pathogen
**Proteobacteria > Gammaproteobacteria > Aeromonadales > Aeromonadaceae**					
237809800	Tolumonas	auensis	DSM 9187	73	Environmental isolate
**Proteobacteria > Gammaproteobacteria > Vibrionales > Vibrionaceae**					
153829420	Vibrio	cholerae	623-39	70	Human pathogen
229526131	Vibrio	cholerae	bv. albensis VL426	70	Human pathogen
15601016	Vibrio	cholerae	O1 biovar El Tor str. N16961	70	Human pathogen
297579636	Vibrio	cholerae	RC385	70	Human pathogen
254225972	Vibrio	cholerae	V51	70	Human pathogen
269959501	Vibrio	harveyi	1DA3	70	Fish pathogen
260773763	Vibrio	metschnikovii	CIP 69.14	71	Human pathogen
262174029	Vibrio	mimicus	MB-451	71	Animal/human pathogen
262164843	Vibrio	mimicus	VM223	71	Animal/human pathogen
258627162	Vibrio	mimicus	VM603	71	Animal/human pathogen
149187852	Vibrio	shilonii	AK1	70	Coral pathogen
86144666	Vibrio	sp	MED222	68	Sea environmental bacterium
261212560	Vibrio	sp	RC341	71	Sea environmental bacterium
161501860	Vibrio	vulnificus	CMCP6	70	Fish pathogen
161486676	Vibrio	vulnificus	YJ016	70	Fish pathogen
148974110	Vibrionales	bacterium	SWAT-3	68	Sea environmental bacterium
54301951	Photobacterium	profundum	SS9	73	Sea environmental bacterium
**Firmicutes > Erysipelotrichi > Erysipelotrichales > Erysipelotrichaceae**					
223983523	Holdemania	filiformis	DSM 12042	61	Human gastrointestinal commensal

283768366	Bulleidia	extructa	W1219	60	Human oral commensal
259504264	Erysipelothrix	rhusiopathiae	ATCC 19414	58	Animal/human enteropathogen
309776791	Erysipelotrichaceae	bacterium	3_1_53	63	Human gastrointestinal commensal
**Firmicutes > Bacillales**					
289550848	Staphylococcus	lugdunensis	HKU09-01	56	Human commensal
**Firmicutes > Clostridia > Clostridiales > Clostridiaceae**					
225386559	Clostridium	asparagiforme	DSM 15981	65	Human commensal
187932596	Clostridium	botulinum	B str. Eklund 17B	76	Presumptive human pathogen
251779368	Clostridium	botulinum	E1 str. 'BoNT E Beluga'	76	Human pathogen
188588544	Clostridium	botulinum	E3 str. Alaska E43	76	Human pathogen
182419279	Clostridium	butyricum	5521	74	Human pathogen
237668726	Clostridium	butyricum	E4	74	Human pathogen
110800572	Clostridium	perfringens	ATCC 13124	74	Human pathogen
182624139	Clostridium	perfringens	D str. JGS1721	74	Human pathogen
168204693	Clostridium	perfringens	E str. JGS1987	74	Human pathogen
168217215	Clostridium	perfringens	NCTC 8239	74	Human pathogen
167766408	Clostridium	sp	SS2/1	59	Animal-associated habitat
**Firmicutes > Clostridia > Clostridiales > Ruminococcaceae**					
154505032	Ruminococcus	gnavus	ATCC 29149	60	Human gastrointestinal commensal
**Firmicutes > Clostridia > Clostridiales**					
168333915	Epulopiscium	sp	N.t. morphotype B'	59	Marine fish commensal
168333356	Epulopiscium	sp	N.t. morphotype B'	68	Marine fish commensal
**Firmicutes > Clostridia > Clostridiales > Clostridiales Family XI, Incertae sedis**					
256545428	Anaerococcus	vaginalis	ATCC 51170	58	Human vaginal commensal
167772710	Anaerotruncus	colihominis	DSM 17241	67	Human colonic commensal
**Firmicutes > Clostridia > Clostridiales > Lachnospiraceae**					
229823912	Catonella	morbi	ATCC 51271	63	Human periodontitis
291520989	Coprococcus	catus	GD/7	60	Rarely pathogenic in humans
**Firmicutes > Thermoanaerobacteriales > Thermoanabacteriacea**					
297543834	Thermoanaerobacte	mathranii	subsp. mathranii str. A3	72	Hot spring isolate
**Firmicutes > Lactobacillales > Enterococcaceae**					
257415739	Enterococcus	faecalis	AR01/DG	63	MDR bacterium from dog wound
229550381	Enterococcus	faecalis	ATCC 29200	63	Human urogenital secretions
256618709	Enterococcus	faecalis	ATCC 4200	63	Isolated from human blood
257089537	Enterococcus	faecalis	CH188	63	MDR bacterium
257085614	Enterococcus	faecalis	Fly1	63	Fruit-fly commensal
256965474	Enterococcus	faecalis	HIP11704	63	Clinical isolate, vancomycin resistant
256962279	Enterococcus	faecalis	Merz96	63	MDR from human blood
255976195	Enterococcus	faecalis	T2	63	MDR from human urinary tract infection
256762133	Enterococcus	faecalis	T3	63	MDR from human urinary tract infection
227518380	Enterococcus	faecalis	TX0104	63	Human endocarditis
311293279	Enterococcus	faecalis	TX0470	63	Presumptive human pathogen
229546196	Enterococcus	faecalis	TX1322	63	Presumptive human pathogen
29375701	Enterococcus	faecalis	V583	66	Human pathogen
257887800	Enterococcus	faecium	1,141,733	66	Human pathogen
257898922	Enterococcus	faecium	Com15	67	Human feces from healthy adult
69247534	Enterococcus	faecium	DO	67	Human pathogen
293553854	Enterococcus	faecium	E1039	66	Human stool isolate
293569231	Enterococcus	faecium	E1071	67	Hospitalized human feces
293379159	Enterococcus	faecium	PC4.1	66	Human pathogen
**Firmicutes > n.a. > Lactobacillales > Carnobacteriaceae**					
259046734	Granulicatella	adiacens	ATCC 49175	69	Human pathogen
260583788	Granulicatella	elegans	ATCC 700633	69	Human pathogen
**Firmicutes > n.a. > Lactobacillales > Lactobacillaceae**					
116496161	Lactobacillus	casei	ATCC 334	64	Human commensal
256847616	Lactobacillus	coleohominis	101-4-CHN	57	Human commensal
227879283	Lactobacillus	crispatus	JV-V01	58	Human commensal
239630599	Lactobacillus	paracasei	subsp. paracasei 8700:02:00	64	Human commensal
258509728	Lactobacillus	rhamnosus	GG	62	Human commensal
199598619	Lactobacillus	rhamnosus	HN001	52	Human commensal
199599397	Lactobacillus	rhamnosus	HN001	62	Human commensal
**Firmicutes > n.a. > Lactobacillales > Streptococcaceae**					
22537939	Streptococcus	agalactiae	2603V/R	58	Human pathogen
25011880	Streptococcus	agalactiae	NEM316	58	Human pathogen
288906297	Streptococcus	gallolyticus	UCN34	56	Animal/human pathogen
306834473	Streptococcus	bovis	ATCC 700338	56	Animal/human pathogen
251783397	Streptococcus	dysgalactiae	subsp. equisimilis GGS 124	57	Human pathogen
225867766	Streptococcus	equi	subsp. zooepidemicus	57	Animal/human pathogen
195977322	Streptococcus	equi	subsp. zooepidemicus MGCS10565	57	Animal/human pathogen
289167112	Streptococcus	mitis	B6	59	Human respiratory tract commensal
307709873	Streptococcus	mitis	SK564	59	Human respiratory tract commensal
307705704	Streptococcus	mitis	SK597	58	Human respiratory tract commensal
24378793	Streptococcus	mutans	UA159	58	Dental caries pathogen
168492452	Streptococcus	pneumoniae	CDC0288-04	58	Human pathogen
168494649	Streptococcus	pneumoniae	CDC3059-06	58	Human pathogen
194398474	Streptococcus	pneumoniae	G54	58	Human pathogen
149007820	Streptococcus	pneumoniae	SP18-BS74	58	Human pathogen
148989728	Streptococcus	pneumoniae	SP6-BS73	58	Human pathogen
15901852	Streptococcus	pneumoniae	TIGR4	58	Human pathogen
15674387	Streptococcus	pyogenes	M1 GAS	57	Human pathogen
94993558	Streptococcus	pyogenes	MGAS10750	57	Human pathogen
21909678	Streptococcus	pyogenes	MGAS315	56	Human pathogen
125718864	Streptococcus	sanguinis	SK36	58	Human pathogen
146319709	Streptococcus	suis	05ZYH33	57	Swine/zoonotic pathogen
222153816	Streptococcus	uberis	0140J	58	Bovine mastitis
311100397	Streptococcus	vestibularis	F0396	57	Human oral commensal
**n.a. > n.a. > Lactobacillales > n.a.**					
300174099	Leuconostoc	gasicomitatum	LMG 18811	62	Food spoilage
116619070	Leuconostoc	mesenteroides	subsp. mesenteroides ATCC 8293	63	Probiotic
**Actinobacteria > Coriobacteridae > Coriobacteriales > Coriobacterinae/Coriobacteriaceae**					
257784134	Atopobium	parvulum	DSM 20469	60	Human oral commensal, halitosis
221195328	Atopobium	rimae	ATCC 49626	54	Human oral commensal, periodontitis
227516887	Atopobium	vaginae	DSM 15829	56	Human vaginal abnormal flora
303232906	Atopobium	vaginae	PB189-T1-4	56	Human vaginal abnormal flora
**Fusobacteria > n.a. > Fusobacteriales > Fusobacteriaceae**					
262039690	Leptotrichia	goodfellowii	F0264	63	Human oral commensal

260891058	Leptotrichia	hofstadii	F0254	62	Human oral commensal
269118772	Sebaldella	termitidis	ATCC 33386	75	Termes gastrointestinal commensal
269124072	Streptobacillus	moniliformis	DSM 12112	64	Rat-bite fever
**Spirochaetes > n.a. > Spirochaetales > Spirochaetaceae**					
302336850	Spirochaeta	smaragdinae	DSM 11293	72	Environmental isolate
