# Supplementary material for: The UlaG protein family defines novel structural and functional motifs grafted on an ancient RNase fold
Source: BMC Evol Biol. 2011 Sep 26;11:273. doi: 10.1186/1471-2148-11-273 (PMC3219644; doi:10.1186/1471-2148-11-273)
Supplement: Additional file 13 — Table S2. Homology modeling, interfaces, and geometry validation of UlaGL homology models generated with MODELLER-9v8. [file 1471-2148-11-273-S13.DOC]

**Additional file 13.**

**Table S2**. Evaluation of UlaG hexamer comparative models calculated with MODELLER-9v8.

|  |  |  |  |  | Ramachandran | |
| --- | --- | --- | --- | --- | --- | --- |
| UlaGL | Z-DOPE | ModTie | Clash | Rotamer outlier, % | Outlier, % | Favored, % |
| *E. coli* (PDB ID 2wym) | -2.096 | -2.934 | 8.29 | 1.56 | 0.00 | 97.05 |
| *V. cholerae* (PDB ID 3bv6) | -1.836 | -3.807 | 7.62 | 1.12 | 0.00 | 99.05 |
| S. enterica serovar Typhi | -1.509 | -3.450 | 81.09 | 0.05 | 0.38 | 97.40 |
| *C. botulinum* | -1.680 | -2.866 | 79.09 | 2.84 | 0.19 | 96.64 |
| *Y. intermedia* | -1.565 | -3.231 | 67.21 | 0.00 | 0.09 | 97.69 |
| *S. pyogenes* | -1.204 | -3.133 | 98.37 | 0.00 | 0.14 | 96.81 |
| *A. vaginae* | -1.255 | -3.360 | 90.04 | 0.05 | 0.14 | 97.82 |

Z-DOPE, ModTie, and MolProbity results for each of the hexamer comparative models are compared. All models were subjected to side chain refinement by SCWRL4 including the *E. coli* and *V. cholerae* UlaGL hexamers, a control based on the crystal structures.
