# Supplementary material for: The UlaG protein family defines novel structural and functional motifs grafted on an ancient RNase fold
Source: BMC Evol Biol. 2011 Sep 26;11:273. doi: 10.1186/1471-2148-11-273 (PMC3219644; doi:10.1186/1471-2148-11-273)
Supplement: Additional file 14 — Table S3. Analysis of buried contact area by PISA of reference structures and homology models generated with MODELLER-9v8. [file 1471-2148-11-273-S14.DOC]

**Additional file 14.**

**Table S3**. Evaluation of buried interfaces by PISA (*1*) from UlaG hexamer comparative models calculated with MODELLER-9v8. Interfaces 1 and 2 refer to the largest and second largest interfaces, respectively, between UlaGL protomers in the hexamer.

| UlaGL | Interface | BSA | –ΔG | HB | SB |
| --- | --- | --- | --- | --- | --- |
|  |  | *Å^2^* | *kcal/mol* |  |  |
| *E. coli* (PDB ID 2wym) | 1 | 3050 | 39 | 45 | 7 |
|  | 2 | 1220 | 3 | 24 | 11 |
| *V. cholerae* (PDB ID 3bv6) | 1 | 4000 | 44 | 58 | 12 |
|  | 2 | 1350 | 1 | 39 | 18 |
| *S. enterica* subsp. Typhi | 1 | 4000 | 66 | 24 | 10 |
|  | 2 | 1200 | 2 | 16 | 6 |
| *C. botulinum* | 1 | 4400 | 62 | 33 | 15 |
|  | 2 | 1260 | 2 | 6 | 13 |
| *Y. intermedia* | 1 | 4600 | 59 | 26 | 6 |
|  | 2 | 1400 | 1 | 22 | 8 |
| *S. pyogenes* | 1 | 4500 | 53 | 33 | 13 |
|  | 2 | 1270 | 3 | 14 | 15 |
| *A. vaginae* | 1 | 4670 | 60 | 14 | 8 |
|  | 2 | 1180 | 6 | 19 | 16 |

Buried surface area (BSA), minus interface free energy (–ΔG), number of hydrogen bonds (HB), and number of salt bridges (SB) results extracted from PISA for each of the hexamer comparative models are compared, including the *E. coli* and *V. cholerae* UlaGL hexamers, a control based on the crystal structures. It is noticeable that the BSA, –ΔG, and SB for the comparative models are within reasonable approximation to the reference crystal structures, whereas the HB are systematically fewer, likely because of their directional nature; since no restraints were applied to specific interfacial contacts HB is a lower bound consistent with the modeling protocols. The number of potential HB could have been much improved by manual refinement of the models.

*1*. Krissinel E, Henrick K: Inference of macromolecular assemblies from crystalline state. *J Mol Biol* 2007, 372(3):774-797.
